# Photocatalytic CO-releasing spray hydrogel for in situ postoperative cancer treatment

**DOI:** 10.1016/j.bioactmat.2025.07.024

**Published:** 2025-08-12

**Authors:** Zaiyan Wang, Jianxiang Zhu, Bobin Mi, Ming Ni, Yuming Xue, Yiling Deng, Lu Chen, Xiangyang Xu, Xiaoyan Li, Guohui Liu, Tao Yu

**Affiliations:** a Department of Pulmonary and Critical Care Medicine, Shanghai University of Medicine & Health Sciences Affiliated Zhoupu Hospital, Shanghai, People's Republic of China; b Department of Orthopaedics, First Affiliated Hospital of Huzhou University, Huzhou, Zhejiang Province, People's Republic of China; c Department of Orthopaedics, Ruijin Hospital, Shanghai Jiao Tong University School of Medicine, Shanghai, People's Republic of China; d Department of Orthopaedics, Ruijin-Hainan Hospital, Shanghai Jiao Tong University School of Medicine (Hainan Boao Research Hospital), Qionghai, Hainan Province, People's Republic of China; e Department of Orthopedics, Union Hospital, Tongji Medical College, Huazhong University of Science and Technology, Wuhan, Hubei Province, People's Republic of China

**Keywords:** Postoperative treatment strategy, Gas therapy, Hydrogels, Redox balance, Nano-photocatalyst

## Abstract

The high invasiveness and metastatic potential of breast cancer increase the risk of postoperative recurrence. To address this issue, a composite hydrogel drug delivery system based on sodium alginate (SA) has been developed for the in situ release of carbon monoxide (CO) at the tumor resection site. This system aims to enhance the effectiveness of chemotherapy and improve the clearance of residual tumor cells after surgery, thereby preventing tumor recurrence and metastasis. A gold nanoparticle-modified g-C_3_N_4_ nanophotocatalyst (C_3_N_4_/Au) has been designed to convert CO_2_ within the tumor into CO under visible light irradiation. The C_3_N_4_/Au is loaded into an SA hydrogel and applied to the postoperative incision in a spray form. The rapid crosslinking reaction between SA and Ca^2+^ forms a network structure, enabling precise drug delivery. CO is generated in situ in the postoperative tumor tissue under light stimulation and is combined with folic acid (FA) modified doxorubicin (DOX) micelles (FA@DM) to achieve effective synergy between CO therapy and chemotherapy. The composite hydrogel drug delivery system not only increases the concentration of CO within the tumor and reduces systemic toxicity but also enhances the effectiveness of chemotherapy by increasing oxidative stress within tumor cells. It provides a new and safe strategy for efficient and precise postoperative tumor treatment, reducing the risk of tumor recurrence and metastasis.

## Highlights

• C3N4/Au nanophotocatalyst enhances CO production efficiency.
• SA hydrogel enables precise drug delivery at postoperative sites.
• CO therapy combined with FA@DM achieves gas-chemo synergy.

## Graphical abstract

**Figure:**
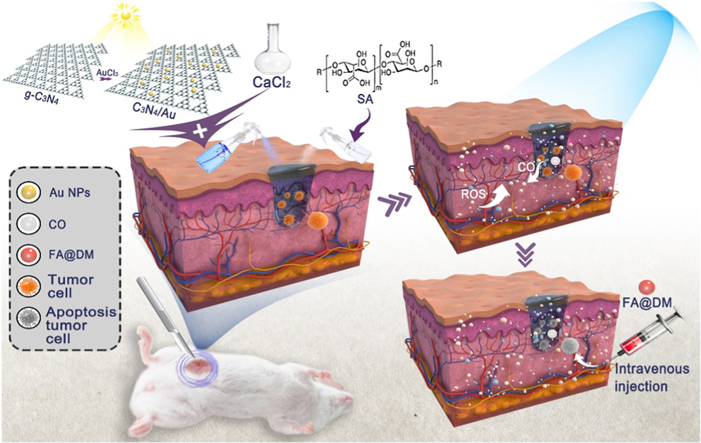

## 1 Introduction

Malignant tumors have become one of the major diseases that severely threaten human health and life globally. Currently, the traditional treatment modalities for tumors mainly include surgery, radiotherapy, and chemotherapy [1]. Among them, surgical treatment is the most common and effective method. However, surgery typically can only remove macroscopically visible tumor tissues and is unable to completely eliminate microscopic tumor cells that have infiltrated normal tissues. According to relevant studies, more than 80 % of patients still need to undergo chemotherapy after surgery [2]. Chemotherapy, which involves the systemic administration of chemical agents, can eliminate tumor cells to some extent and thus inhibit tumor recurrence and metastasis [3]. Regrettably, chemotherapeutic drugs lack the ability to precisely identify tumor cells. While killing cancer cells, they also cause significant damage to normal cells. Moreover, chemotherapy faces numerous issues, such as multidrug resistance, which severely limit its therapeutic efficacy [4]. Therefore, the development of new, highly efficient, and low-toxicity postoperative treatment strategies is particularly urgent. Such advances are crucial for improving the prognosis of tumor patients and enhancing their quality of life.

In recent years, as the physiological regulatory functions of gas molecules in cancer development have been revealed, gas therapy has attracted increasing attention from researchers [5,6]. Due to its low toxicity and high efficiency in the body, it is considered a safe and effective “green” cancer treatment method. Endogenous gas molecules, such as nitric oxide (NO) [7], carbon monoxide (CO) [8,9], and hydrogen sulfide (H_2_S) [10], play key roles in the body's physiological and pathological processes. They participate in various biological processes, including cell signaling, regulation of vascular tension, and mediation of immune responses. Compared with traditional drug treatments, gas molecules have the advantages of small molecular weight, fast diffusion speed, and easy penetration of biological barriers, allowing them to rapidly reach the target site and achieve efficient biological effects. Notably, CO can interfere with the antioxidant defense systems of cancer cells, disrupting the redox balance within tumor cells and enhancing their sensitivity to oxidative stress damage induced by chemotherapeutic drugs [[11], [12], [13]]. Researchers have developed various carbon monoxide-releasing molecules (CORMs) to achieve slow or targeted release of carbon monoxide in the body [14]. However, most CORMs have a short half-life and are quickly metabolized and decomposed, making it difficult to maintain a stable concentration of CO and fully exert their therapeutic effects [15]. Due to the intensification of the greenhouse effect, the conversion and utilization of carbon dioxide (CO_2_) have become popular, with CO being one of the reduction products. Numerous studies are exploring efficient CO_2_ photocatalytic reduction materials, including metal nanoparticles [16], metal-organic frameworks (MOFs) [17], and two-dimensional materials [18]. Among these, Graphitic carbon nitride (g-C_3_N_4_) is a two-dimensional photocatalyst with good biocompatibility, featuring a large specific surface area and an appropriate bandgap structure [19]. This allows it to respond in the visible light range, facilitating the separation and transfer of photogenerated charges, and is widely used in photocatalytic water splitting and other fields. Modifying g-C_3_N_4_ can change the selectivity and yield of its photocatalytic products. For example, loading gold or silver nanoparticles on the surface of g-C_3_N_4_ broadens its light absorption range and acts as an electron trap, effectively promoting the separation of photogenerated charges, inhibiting charge recombination, and thereby significantly enhancing its photocatalytic activity for CO production [[20], [21], [22]]. Additionally, tumor cells have a higher concentration of HCO_3_^−^ and a lower pH value compared to the atmospheric environment (pCO_2_ ≈ 0.2 mmHg), resulting in a higher concentration of CO_2_ in tumor tissues (pCO_2_ ≈ 80 mmHg) [23]. Therefore, using photocatalysts to continuously and controllably produce CO in the body can improve the effectiveness of chemotherapy while enhancing its safety.

Systemic drug administration is not conducive to the enrichment of photocatalysts in tumor regions. Moreover, the low tissue-penetrating ability of blue light makes it difficult to irradiate the photocatalysts distributed within the tissues. For postoperative tumor therapy, the in situ drug delivery strategy involves directly applying the drug or drug-loaded carrier onto the surgical wound surface, allowing irradiation the site directly. This approach circumvents the limitation of insufficient tissue penetration by visible or near-infrared light. [24]. Compared to traditional methods, in situ administration increases the concentration of CO at the tumor site while avoiding its widespread distribution throughout the body. This significantly reduces unnecessary damage to normal tissues and lowers systemic toxic reactions [25]. Hydrogels, as three-dimensional network-structured polymeric materials, possess good biocompatibility and can simulate the microenvironment of human tissues, providing a suitable environment for cell adhesion and proliferation [26]. Their unique physicochemical properties enable the effective loading of various drugs, bioactive molecules, and cells, achieving controlled and sustained drug release [27,28]. Among them, injectable hydrogels can rapidly gelate through physical or chemical methods after injection. This not only prolongs the residence time of drugs at the tumor site but also enables minimally invasive drug delivery, thus having a broader range of applications [[29], [30], [31]]. Sodium alginate (SA) is a natural polysaccharide extracted from brown algae such as kelp or Sargassum, with good biodegradability and biocompatibility [[32], [33], [34]]. It can gradually degrade into non-toxic small molecules in the body, which are then metabolized and excreted. SA undergoes rapid and mild crosslinking reactions with calcium ions to form stable network hydrogel structures. This in situ hydrogel formation characteristic facilitates in situ drug delivery at the surgical site. Compared to other injectable hydrogels, such as those that are photocross-linked or based on Schiff-base reactions, SA hydrogels present the advantage of eliminating the need for initiators or supplementary chemical modifications. Therefore, their composition and properties are more readily adjustable [35]. The photocatalyst can be loaded into either calcium chloride (CaCl_2_) solution or SA solution. These two precursors can be swiftly combined at the tumor resection site using techniques such as spraying [36,37]. The hydrogel formed by the crosslinking of SA with calcium ions can cover the wound surface, acting as a physical barrier to effectively prevent the invasion of external bacteria and reduce the risk of postoperative infection [38]. Additionally, the loaded photocatalyst can continuously produce CO under light exposure. Although light irradiation is limited to the surface of the surgical incision, CO is capable of penetrating tissues via free diffusion and subsequently entering tumor cells, thereby increasing their sensitivity to chemotherapy. This sprayable hydrogel drug delivery system is simple and convenient to operate, allowing for immediate preparation and application at the surgical site. It serves as a powerful tool for postoperative adjuvant treatment of tumors.

**Scheme 1:**
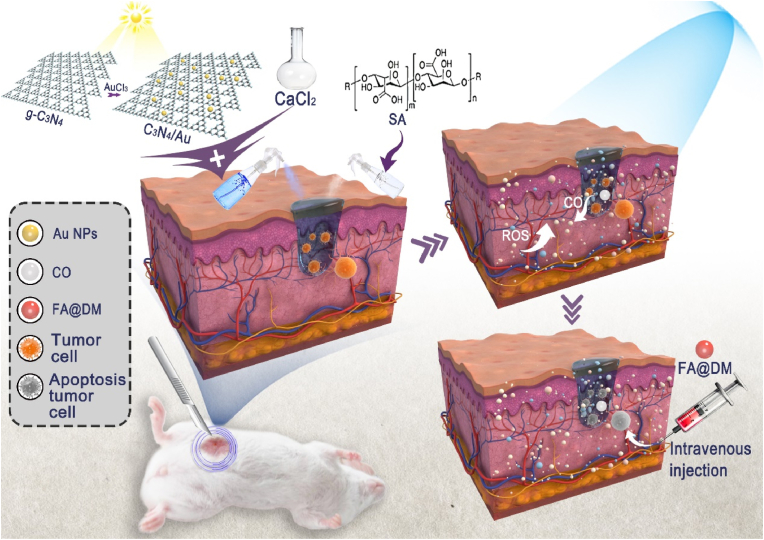
Schematic diagram of in situ tumor-generated CO sensitized chemotherapy by spray of nano-photocatalyst C_3_N_4_/Au.

In this study, a sprayable hydrogel composite system loaded with a photocatalyst was constructed and applied to a postoperative breast cancer model to enhance chemotherapy efficacy and prevent tumor recurrence. C_3_N_4_ nanoparticles were modified with Au nanoparticles to prepare the photocatalyst (C_3_N_4_/Au), which was then mixed with a calcium chloride solution. An in situ hydrogel C_3_N_4_/Au/SA was formed by spraying with an SA solution. The C_3_N_4_/Au/SA adheres to the postoperative tumor wound surface. Upon light stimulation, CO is generated in situ within the postoperative tumor tissue, which increases the oxidative stress in tumor cells. Subsequently, folic acid (FA)-modified doxorubicin (DOX) micelles (FA@DM) are intravenously injected, delivering DOX to breast cancer cells that overexpress FA receptors [39], synergistically enhancing the chemotherapy effect with CO (Scheme 1). This strategy significantly improves the convenience and controllability of using CO gas for postoperative tumor treatment, achieving the goal of combining gas therapy with chemotherapy to inhibit postoperative recurrence and metastasis of malignant tumors.

## 2 Materials and methods

### 2.1 Materials

Melamine, chloroauric acid trihydrate, 3-nitro-1,8-naphthalic anhydride, 4-(2-aminoethyl)morpholine, 4-(2-aminoethyl)morpholine, dimethyl sulfoxide, 5,5-dimethyl-1-pyrroline-N-oxide (DMPO) and 2-(tert-butoxycarbonyl)-2-methyl-3,4-dihydro-2H-pyrrole 1-oxide (BMPO) were purchased from Shanghai Aladdin Biochemical Technology Co., Ltd. 2,2,5,5-Tetramethyl-2,5-dihydro-1H-pyrrole-3-carboxamide (TPC), SA, CaCl_2_ and DOX were purchased from Shanghai Macklin Biochemical Technology Co., Ltd. Hydrochloric acid (HCl) was purchased from Zhejiang Sanying Chemical Reagent Co., Ltd. Anhydrous methanol and anhydrous ethanol were purchased from Hangzhou Gaojing Fine Chemical Co., Ltd. 1,2-distearoyl-sn-glycero-3-phosphoethanolamine-N-polyethyleneglycol-folate (DSPE-PEG_2000_-FA), 1,2-distearoyl-sn-glycero-3-phosphoethanolamine-N-polyethyleneglycol-3-dimethyl-1-(4-sulfonatobutyl)benz[e]indolinium (DSPE-PEG_2000_-ICG) and 1,2-distearoyl-sn-glycero-3-phosphoethanolamine-N-polyethyleneglycol (DSPE-PEG_2000_) were sourced from Shanghai Pengshuo Biotechnology Co., Ltd. CCK-8 kit, Annexin V-FITC/PI detection kit, reactive oxygen species detection kit, 2′,7′-Dichlorodihydrofluorescein diacetate (DCFH-DA) and mitochondrial membrane potential detection kit (JC-1) were obtained from Shanghai Beyotime Biotechnology Co., Ltd.

### 2.2 Material characterization

The transmission electron microscope (JEM-2100Plus, JEOL) was used to observe the microscopic morphology, lattice structure, and elemental distribution of the material. The ultraviolet–visible spectrophotometer (U-3900H, Hitachi) was used to determine the absorption spectra of the material in the ultraviolet–visible region. The X-ray diffractometer (D8-Advance, Bruker) was used to analyze the crystal structure of the material and obtain the X-ray powder diffractometer (XRD) patterns. X-ray photoelectron spectroscopy (XPS) was used to characterize the surface chemical properties and elemental chemical states of the materials using an X-ray photoelectron spectrometer (K-Alpha, Thermo Fisher). The ultraviolet–visible (UV–Vis) spectra of the materials were measured by an ultraviolet–visible spectrophotometer (TU-1901, Persee). The Fourier transform infrared (FTIR) spectrometer (Nicolet iS50, Thermo Fisher) was used to conduct the infrared spectroscopy test on the material. For this test, the KBr pellet method was adopted; the sample was mixed and ground with KBr and then pelletized for determination. The nuclear magnetic resonance spectrometer (FTNMR Digital, Bruker) and the high-resolution mass spectrometer (JMS-T100LP AccuTOF, JEOL) were used to characterize the structure and molecular weight of the compounds. A universal material testing machine (5943, Instron) was used to characterize the tensile and compressive properties of the hydrogel. A rotational rheometer (Mars60, Haake) was used to characterize the rheological properties of the hydrogel.

### 2.3 Synthesis of g-C_3_N_4_

3 g of melamine was placed in an alumina crucible, heated to 550 °C in a muffle furnace at a heating rate of 3 °C/min and maintained for 4 h. After cooling to room temperature, the resulting powder was ground and then dispersed in 100 mL of aqueous HCl solution (5 mol/L) under reflux for 24 h. The product was filtered and washed repeatedly with deionized water until neutrality was achieved. After vacuum drying, a pale-yellow g-C_3_N_4_ powder was obtained.

### 2.4 Synthesis of C_3_N_4_/Au

1 g of g-C_3_N_4_ powder was dispersed in a mixed solvent of 70 mL deionized water and 30 mL anhydrous methanol. Subsequently, 1 mL of an aqueous AuCl_3_ solution (25 mg/mL) was added. The mixture was irradiated under continuous stirring using a 300 W xenon lamp for 3 h, during which the solid phase gradually turned pale purple. The resulting precipitate was collected by centrifugation, washed three times with deionized water, and finally freeze-dried to obtain the C_3_N_4_/Au powder.

### 2.5 Preparation of C_3_N_4_/Au/SA hydrogel

20 mg of C_3_N_4_/Au was mixed with 10 mL of a 2 mol/L aqueous CaCl_2_ solution and dispersed evenly using an ultrasonic processor to obtain dispersion A. 20 mg of SA was dissolved in 1 mL of deionized water under heating to obtain solution B. Dispersions A and B were mixed at a volume ratio of 1:2 by spraying to obtain the C_3_N_4_/Au/SA hydrogel.

### 2.6 Preparation of DOX micelles (DM)

5 mg of DOX were mixed with 25 mg of DSPE-PEG_2000_ and dissolved in 2 mL of DMSO. The mixture was then dialyzed against deionized water for 24 h to obtain DM.

### 2.7 Preparation of FA@DM

5 mg of DOX, 5 mg of DSPE-PEG_2000_-FA, and 25 mg of DSPE-PEG_2000_ were mixed and dissolved in 2 mL of DMSO. The mixture was then dialyzed against deionized water for 24 h to obtain FA@DM.

### 2.8 Preparation of indocyanine green (ICG) loaded DOX micelles (DM@ICG)

1 mg of DOX, 0.25 mg of DSPE-PEG_2000_-ICG, and 5 mg of DSPE-PEG_2000_ were mixed and dissolved in 1 mL of DMSO. The mixture was then dialyzed against deionized water for 24 h to obtain DM@ICG.

### 2.9 Preparation of FA modified DM@ICG (FA@DM@ICG)

1 mg of DOX, 1 mg of DSPE-PEG_2000_-FA, 0.25 mg of DSPE-PEG_2000_-ICG, and 5 mg of DSPE-PEG_2000_ were mixed and dissolved in 1 mL of DMSO. The mixture was then dialyzed against deionized water for 24 h to obtain FA@DM@ICG.

### 2.10 Synthesis of 2-(2-Morpholin-4-ylethyl)-5-nitrobenzo[de]isoquinoline-1,3-dione (LysoFP-NO_2_)

A solution of 4-(2-aminoethyl)morpholine (0.156 g) in 30 mL of ethanol was slowly dropped into a solution of 3-nitro-1,8-naphthalic anhydride (0.243 g) in 50 mL of ethanol, then the mixture was refluxed for 3 h. After filtration, the solvent was removed by rotary evaporation and vacuum drying to obtain LysoFP-NO_2_.

### 2.11 Detection of CO production

For the fluorescence detection method, LysoFP-NO_2_ was dissolved with the aid of 1 % DMSO and added to aqueous dispersions containing g-C_3_N_4_ (5 mg/mL) or C_3_N_4_/Au (5 mg/mL) to achieve a final concentration of 10 μmol/L. The dispersions were then irradiated with 410 nm light-emitting diode (LED) light (100 mW/cm^2^). The fluorescence emission spectra were measured every 1 min of light irradiation using a fluorescence spectrophotometer (FluoroMax-4, HORIBA), with an excitation wavelength of 440 nm.

For the gas chromatography method, 30 mg of g-C_3_N_4_ or C_3_N_4_/Au was used as the catalyst. The photocatalytic products were measured using a photocatalytic system (Labsolar-6A, PerfectLight). A 300 W xenon lamp was used to simulate sunlight irradiation, with the light source positioned 4 cm from the reaction system. Samples were collected and analyzed every hour to monitor the changes in the concentrations of CO and methane (CH_4_) in the system, using a gas chromatograph (GC2002, KE CHUANG).

### 2.12 Detection of reactive oxygen species (ROS)

ROS generated during the photocatalytic process were detected using an electron spin resonance (ESR) spectrometer (EMX PLUS, Bruker). DMPO was used as a spin trap for hydroxyl radicals (·OH), BMPO for superoxide anions (O_2_^−^), and TPC for singlet oxygen (^1^O_2_). The system was irradiated with 410 nm LED light for 5 min or left unirradiated. The presence of ROS was determined by observing the appearance or disappearance of characteristic signal peaks in the ESR spectrum.

### 2.13 Cell culture

RPMI 1640 medium (added with 10 % FBS and 1 % antibodies) was used for 4T1 (triple-negative breast cancer cell line), and L929 (mouse fibroblast cell line) cell culture. All the cell cultures were conducted in a humidified atmosphere (37 °C, 5 % CO_2_).

### 2.14 In vitro CO generation detection

For in vitro CO production, L929 or 4T1 cells were evenly seeded in culture dishes at a density of 10^5^ cells/mL. After incubation for 24 h, different materials were added and incubated for 4 h, followed by light-irradiated and non-irradiated treatments (irradiation time was 30 min, and the light source was a 410 nm LED). The pre-synthesized LysoFP-NO_2_ probe was added and incubated with cells for 30 min. Then, the residual probe was washed away with phosphate buffered solution (PBS), and the fluorescence intensity was analyzed using a laser confocal microscope (C2, Nikon). Five groups were set up in the experiment: (1) Blank, (2) g-C_3_N_4_, (3) C_3_N_4_/Au, (4) g-C_3_N_4_+Light, (5) C_3_N_4_/Au + Light.

The intracellular CO level was quantitatively analyzed by flow cytometry. L929 or 4T1 cells were seeded in six-well plates at a density of 10^5^ cells/mL and then transferred to an incubator for 24 h. Different materials were added to the six-well plates and co-incubated with L929 cells for 6 h. Subsequently, a PBS solution containing the LysoFP-NO_2_ probe (10 μM) was added and co-incubated with the cells for 30 min. The cells were irradiated with a 410 nm LED for 30 min, and then the cells were collected for detection using a flow cytometer (V6B5R3, Advanteon).

### 2.15 Cytocompatibility of hydrogels

The hydrogel was immersed in cell culture medium for 24 h. Then, the leaching solution of the hydrogel was extracted for cell viability determination. L929 cells were seeded into a 96-well plate at a density of 8 × 10^3^ cells per well. After 24 h, the culture medium was replaced with the leaching solution. After co-culturing for 24 or 48 h, 10 μL of the CCK-8 solution was added to each well, and the plate was incubated in the dark for 1 h. The absorbance was measured at 450 nm using a microplate reader.

### 2.16 Cytotoxicity

4T1 cells were seeded into a 96-well plate at a density of 8 × 10^3^ cells per well. After 24 h, each well was treated with different groups. Following a 24 h treatment, 10 μL of the CCK-8 solution was added to each well, and the plate was incubated in the dark for 1 h. The absorbance was measured at 450 nm using a microplate reader.

### 2.17 In vitro detection of ROS

4T1 cells were seeded at a density of 1.5 × 10^5^ cells per well in a six-well plate. After the cells adhered to the surface, the drug was added and incubated for 6 h. Subsequently, DCFH-DA (5 μM) was added and the cells were stained in the dark for 30 min. For the light exposure group, photodynamic treatment was performed (410 nm, 100 mW/cm^2^, 5 min). The cells were then washed three times with PBS and imaged under a confocal microscope or collected for flow cytometry analysis.

### 2.18 Cell apoptosis detection assay

The cell apoptosis detection was performed on cells treated with the Control group, the C_3_N_4_/Au group, and the C_3_N_4_/Au + L group using the Annexin V-FITC/PI double-staining method. The specific experimental procedures were as follows:

4T1 cells were seeded at a density of 1.5 × 10^5^ cells/well in six-well plates. After the cells adhered to the plate and grew to an appropriate density, the culture medium was replaced with fresh medium. According to the experimental group design, the cells were treated with either blank medium or medium containing C_3_N_4_/Au (at a concentration of 50 μg/mL) for 6 h. Subsequently, light irradiation (410 nm, 100 mW/cm^2^, 5 min) was applied to the C_3_N_4_/Au + L group, while the other groups were not subjected to light irradiation. After an additional 48 h of incubation, the medium was aspirated and replaced with 1 mL of PBS to collect the cells. The cells were then centrifuged at 1200 r/min for 3 min. The supernatant was discarded, and the cell pellet was retained. Next, the cell pellet was stained with Annexin V-FITC and PI staining solution and incubated in the dark for 20 min. After incubation, the cells were washed with PBS, resuspended, and filtered, followed by detection using a flow cytometer.

### 2.19 Mitochondrial membrane potential detection assay

4T1 cells were seeded at a density of 1.5 × 10^5^ cells per well in a six-well plate. After the cells adhered and reached an appropriate density, different treatments were applied, and the cells were incubated for 6 h. For the light exposure group, light treatment was performed (410 nm, 100 mW/cm^2^, 5 min). Following this, the cells were further incubated for 24 h. Subsequently, JC-1 staining solution (2 μM) was added, and staining was carried out in the dark for 20 min. Finally, the cells were collected for flow cytometry analysis or confocal imaging.

### 2.20 Cell scratch assay

4T1 cells were added to a six-well plate and incubated at 37 °C with 5 % CO_2_ until the cells formed a confluent monolayer. A sterile 200 μL pipette tip was used to make scratches vertically on the cell surface. Then, the cells were washed three times with PBS to remove cell debris. Finally, the cells were incubated with the g-C_3_N_4_ (50 μg/mL), C_3_N_4_/Au (50 μg/mL) and C_3_N_4_/Au (50 μg/mL) + Light (410 nm, 100 mW/cm^2^, 30 min). Images were taken at 0, 6, 12, and 24 h to observe the scratch-healing situation.

### 2.21 Animals

Female BALB/c mice (6−8 weeks old, about 20 g) were purchased from the SPF Biotechnology Co., Ltd. (Beijing, China). All experiments and animal procedures were approved by the Ethical Committee of Shanghai Pudong New Area Zhoupu Hospital and conducted in accordance with the Guide for Care and Use of Laboratory Animals.

### 2.22 In vivo distribution imaging

A 4T1 tumor-bearing mouse model was constructed. When the tumor volume reached 150 mm^3^, the tumor-bearing mice were randomly divided into two groups. DM@ICG or FA@DM@ICG was administered intravenously to the mice. The in vivo distribution of ICG at different time points was tracked and monitored using a second near-infrared (NIR-II) in vivo imaging system (excitation wavelength: 808 nm, emission filter: 900 nm). After 24 h, the mice were euthanized, and the heart, liver, spleen, lung, kidney, and tumor tissues were harvested for imaging analysis.

### 2.23 In vivo antitumor experiments and immunofluorescence experiments

A 4T1 tumor-bearing mouse model was first established. When the tumor volume reached 100 mm^3^, 80 % of the tumor tissue was resected, and the mice were randomly divided into six groups: Control, C_3_N_4_/Au/SA, C_3_N_4_/Au/SA + L, FA@DM, C_3_N_4_/Au/SA + FA@DM, and C_3_N_4_/Au/SA + FA@DM + L. The specific treatment conditions for each group are as follows: The Control group mice received no treatment after surgery. The C_3_N_4_/Au/SA group and C_3_N_4_/Au/SA + L group mice had the C_3_N_4_/Au solution and SA solution sprayed onto the wound site after surgery. For the C_3_N_4_/Au/SA + L group, the wound site was irradiated with an 18 W LED light (wavelength 410 nm, power density 100 mW/cm^2^) for 5 min after spraying. The FA@DM group mice were administered DOX at a dose of 5 mg/kg via intravenous injection after surgery. The C_3_N_4_/Au/SA + FA@DM group and C_3_N_4_/Au/SA + FA@DM + L group mice had the hydrogel sprayed onto the wound site and were then administered DOX at a dose of 5 mg/kg via intravenous injection after surgery. For the C_3_N_4_/Au/SA + FA@DM + L group, the wound site was irradiated with an 18 W LED light (wavelength 410 nm, power density 100 mW/cm^2^) for 5 min.

The specific hydrogel spraying method is as follows: Firstly, two solutions were prepared: one was a 2 mg/mL C_3_N_4_/Au calcium chloride solution, and the other was a 2 % (w/v) SA solution. The molar concentration of the calcium chloride solution was 2 mol/L. These two solutions were separately loaded into spray bottles for later use. Subsequently, through precise calculations, it was determined that the volume of solution sprayed by the spray bottle each time was 120 μL. Based on this data, the spraying operation was performed at a volume ratio of 1:2. Specifically, immediately after surgery, the C_3_N_4_/Au calcium chloride solution was sprayed onto the postoperative wound site once, followed by two consecutive sprays of the SA solution. During this process, the two solutions rapidly reacted at the postoperative wound site, forming a SA hydrogel containing 0.24 mg of C_3_N_4_/Au.

During the treatment period, the body weight of the mice was measured every two days. On day 21, the mice were euthanized, and the heart, liver, spleen, lung, kidney, and tumor tissues were harvested for further characterization.

After the 21-day treatment, the tumor tissues were collected and processed for paraffin embedding. Deparaffinization with xylene was performed, followed by sectioning and immunohistochemical and immunofluorescence staining of the tissues. The results were observed and analyzed using confocal laser scanning microscopy (CLSM).

### 2.24 In vivo CO diffusion concentration within tumors

A 4T1 tumor-bearing mouse model was established. When the tumor volume reached 100 mm^3^, 80 % of the tumor tissue was resected, and then the mice were randomly divided into 4 groups: Control, C_3_N_4_/Au/S, and two C_3_N_4_/Au/SA + L groups, with 3 mice in each group. The control group received no treatment after surgery. The C3N4/Au/SA group and C3N4/Au/SA + L group mice had the C3N4/Au solution and SA solution sprayed onto the wound site after surgery. The concentrations of the C_3_N_4_/Au and SA solutions and the spraying volumes were the same as those in the antitumor experiment, except a CO probe LysoFP-NO_2_ with a concentration of 10 μg/mL was added to the C_3_N_4_/Au solution. For the two C_3_N_4_/Au/SA + L groups, the wound sites were irradiated with an 18 W LED light (wavelength 410 nm, power density 100 mW/cm^2^) for 2.5 and 5 min after spraying, respectively. 30 min later, the mice were euthanized, and the tumor tissues were collected. The CO concentration in the tumors was quantitatively analyzed by flow cytometry.

### 2.25 Statistical analysis

Significant difference between the two groups was calculated by the two-tailed Student's *t*-test. *p* < 0.05, 0.01, 0.001, and 0.0001 was considered a statistically significant difference and remarked with ∗, ∗∗, ∗∗∗, and ∗∗∗∗, respectively.

## 3 Results and discussion

### 3.1 Synthesis and Characterization of g-C_3_N_4_ and C_3_N_4_/Au

**Fig. 1:**
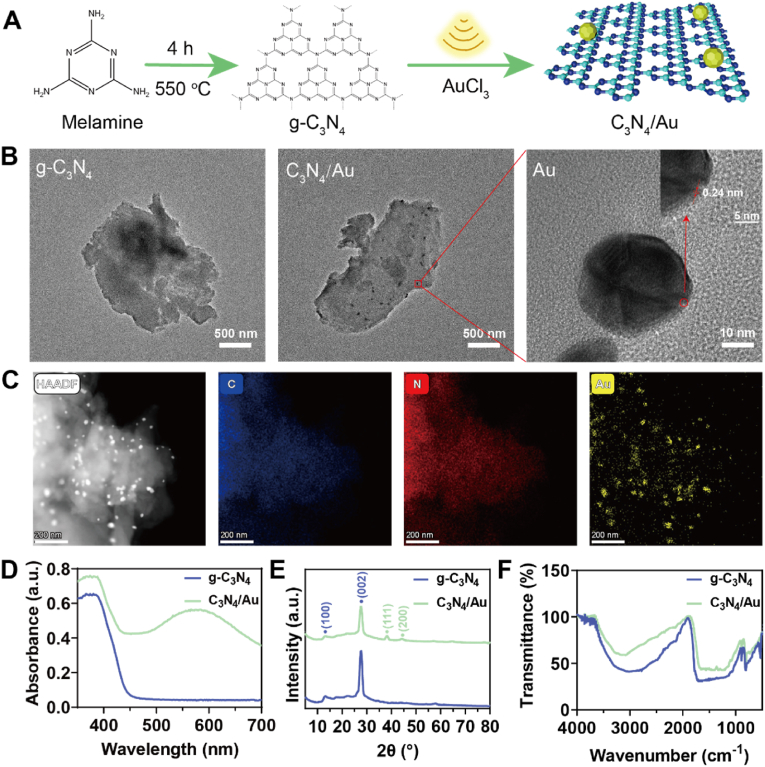
Synthesis and Characterization of C_3_N_4_/Au. **A)** Synthesis Route of C_3_N_4_/Au. **B)** TEM and HRTEM images of g-C_3_N_4_ and C_3_N_4_/Au. **C)** Elemental mapping images of C_3_N_4_/Au. **D)** UV–Vis absorption spectra of g-C_3_N_4_ and C_3_N_4_/Au. **E)** XRD patterns of g-C_3_N_4_ and C_3_N_4_/Au. **F)** FTIR spectra of g-C_3_N_4_ and C_3_N_4_/Au.

The in-situ photodeposition method was employed to load Au nanoparticles (AuNPs) onto the surface of g-C_3_N_4_, thereby enhancing the carrier migration ability of g-C_3_N_4_ and improving the selectivity of CO_2_ reduction to CO. As illustrated in Fig. 1A,g-C_3_N_4_ was successfully synthesized using the high-temperature polycondensation method with melamine as the raw material. Subsequently, Au nanoparticles were loaded onto the surface of g-C_3_N_4_ using the in-situ reduction method, resulting in the preparation of the C_3_N_4_/Au nanophotocatalyst. As shown in Fig. 1B, transmission electron microscopy (TEM) was used to observe the morphology of g-C_3_N_4_ and C_3_N_4_/Au. The prepared g-C_3_N_4_ exhibited an irregular flake-like structure with a diameter of approximately 2 μm. After treatment with AuCl_3_ under light irradiation, distinct small-sized AuNPs were observed on C_3_N_4_/Au. Furthermore, high-resolution transmission electron microscopy (HRTEM) provided an enlarged view of AuNPs, revealing lattice fringes with a spacing of 0.235 nm on C_3_N_4_/Au, corresponding to the (111) plane of Au. Additionally, TEM element mapping precisely displayed the uniform distribution of C, N, and Au elements in the C_3_N_4_/Au composite (Fig. 1C). The Au element is dispersed on the plane composed of C and N, indicating that the AuNPs were evenly dispersed on the surface of g-C_3_N_4_ and formed a stable composite structure. The atomic percentage of Au was 0.41 %, further confirming the successful loading of Au nanoparticles.

The UV–Vis spectrophotometer was used to further characterize g-C_3_N_4_ and C_3_N_4_/Au. Fig. 1D showed that the C_3_N_4_/Au composite exhibited a characteristic peak near 570 nm, with increased absorbance relative to g-C_3_N_4_. This peak is attributed to the surface plasmon resonance absorption of Au nanoparticles, indicating that AuNPs enhance the spectral response range and charge carrier separation efficiency of the nanophotocatalyst. The XRD results, presented in Fig. 1E, confirmed the crystal structure of g-C_3_N_4_ and C_3_N_4_/Au. Both materials displayed diffraction peaks of g-C_3_N_4_ at 2θ angles of 13.2° and 27.6°, corresponding to the (100) and (002) crystal planes. Additionally, C_3_N_4_/Au exhibited new diffraction peaks at 38.3° and 44.4°, corresponding to the (111) and (200) crystal planes of Au, further confirming that the loading of AuNPs did not destroy the crystal structure of g-C_3_N_4_. As shown in Fig. 1F, FTIR spectra indicated that g-C_3_N_4_ and C_3_N_4_/Au possess the same functional groups, with the characteristic peak at 810 cm^−1^ being a typical absorption peak of the triazine heterocycle.

### 3.2 The performance of C_3_N_4_/Au in photocatalytic generation of CO and ROS

The CO fluorescent probe LysoFP-NO_2_ was introduced to measure the CO generation capacity of C_3_N_4_/Au [40]. The synthetic route of LysoFP-NO_2_ is presented in Fig. S2. LysoFP-NO_2_ is a lysosome-targeted fluorescent probe that is based on 3-nitrophthalimide. CO can reduce the nitro group in LysoFP-NO_2_ to a high-fluorescence-intensity amino-functionalized derivative, LysoFP-NH_2_. The characterization of LysoFP-NO_2_ was performed using ^1^H NMR and high-resolution mass spectrometry (HRMS). As shown in Fig. S3, the number and chemical shifts of all hydrogen atoms in the ^1^H NMR spectrum are consistent with the structure of LysoFP-NO_2_. As shown in Fig. S4, the measured mass-to-charge ratio of the sample by HRMS is 356.12505, and the calculated molecular formula is C_18_H_17_N_3_O_5_, which is consistent with [LysoFP-NO_2_ + H]^+^. These results collectively confirm the successful synthesis of LysoFP-NO_2_.

**Fig. 2:**
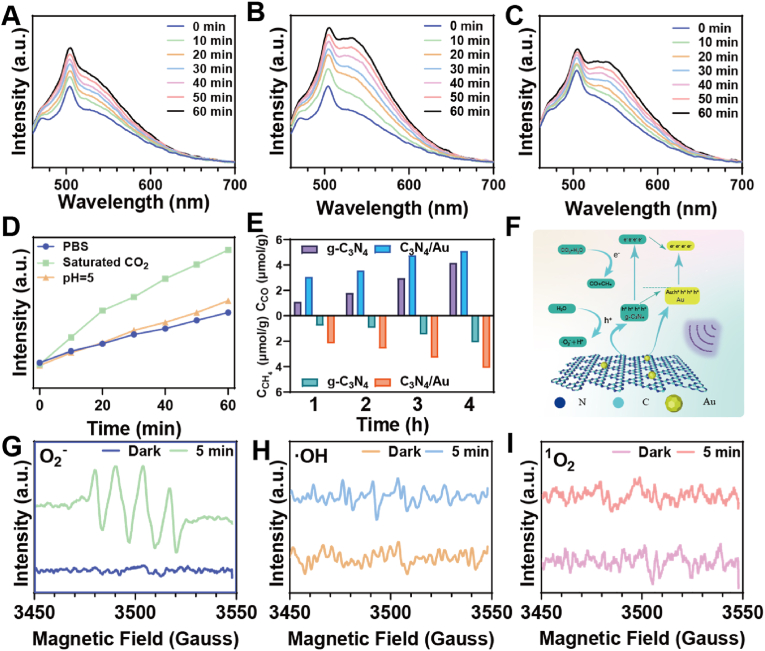
The performance and mechanism of CO generation by photocatalysis of C_3_N_4_/Au. The fluorescence spectra of C_3_N_4_/Au detected by LysoFP-NO_2_, with C_3_N_4_/Au dispersed in **A)** PBS (pH = 7.4), **B)** saturated CO_2_ aqueous solution, and **C)** citrate buffer (pH = 5.0), respectively. **D)** Fluorescence emission intensities at 540 nm of C_3_N_4_/Au detected by LysoFP-NO_2_ changes with light exposure time. **E)** Photocatalytic CO and CH_4_ evolution concentrations of g-C_3_N_4_ and C_3_N_4_/Au changes with light exposure time. **F)** Schematic diagram of the photocatalytic reduction mechanism of C_3_N_4_/Au. **G)** ESR spectra of O_2_^−^ after incubation with C_3_N_4_/Au before and after light exposure. **H)** ESR spectra of ·OH after incubation with C_3_N_4_/Au before and after light exposure. **I)** ESR spectra of ^1^O_2_ after incubation with C_3_N_4_/Au before and after light exposure.

The fluorescence change of the LysoFP-NO_2_ probe was used to evaluate the ability of C_3_N_4_/Au to photocatalytically generate CO in PBS (pH = 7.4), saturated CO_2_ solution and citrate buffer solution (pH = 5). As shown in Fig. 2A–C, under 410 nm LED irradiation, the fluorescence intensity of the probe increased with the increase of irradiation time, indicating that C_3_N_4_/Au has the ability to photocatalytically generate CO. In addition, the comparison of the CO generation rates in the three solutions is shown in Fig. 2D. It can be observed that C_3_N_4_/Au can catalyze the generation of more CO in the saturated CO_2_ solution, while the amount of CO generated in the acidic environment is close to that in the neutral environment. This proved that the CO_2_ concentration affected the photocatalytic efficiency. It also indicated that in the tumor environment with a high CO_2_ concentration, C_3_N_4_/Au can generate CO efficiently for chemotherapy sensitization.

To further verify the photocatalytic products, the material was illuminated with a xenon lamp to simulate sunlight in a sealed system, and the composition and quantity of the generated gases were analyzed using gas chromatography. As shown in Fig. 2E, the primary products of CO_2_ photocatalytic reduction by C_3_N_4_/Au are CO and CH_4_, with CO being produced at a higher rate than CH_4_ [41]. In comparison to g-C_3_N_4_, C_3_N_4_/Au demonstrated significantly higher rates of CO and CH_4_ production, confirming that the incorporation of AuNPs enhanced the photocatalytic efficiency of g-C_3_N_4_. Methane possesses anti-inflammatory activity and has been used in research on the treatment of intestinal diseases [42]. However, its reactivity is much lower than CO, so its effect on tumors as a by-product is negligible [43]. The mechanism of CO_2_ photocatalytic reduction by C_3_N_4_/Au is illustrated in Fig. 2F. Under 410 nm irradiation, g-C_3_N_4_ primarily facilitates the photodecomposition of water into hydrogen and oxygen. Concurrently, it exhibits the capability to reduce a minor fraction of carbon dioxide, resulting in the production of CO and CH_4_. The introduction of AuNPs not only broadens the visible light response range of g-C_3_N_4_ but also improves the carrier separation efficiency, enhancing the selectivity and rate of CO generation.

Furthermore, ESR was employed to detect the reactive oxygen species produced during the photocatalytic process, thereby corroborating the mechanism of CO_2_ photocatalytic reduction. Fig. 2G–I shows significant characteristic peak changes for O_2_^-^, a faint characteristic peak for ·OH, and no characteristic peak for ^1^O_2_. This suggests that during the photocatalytic process of C_3_N_4_/Au, O_2_^-^ and ·OH may participate in the reduction of CO_2_. Specifically, O_2_^-^ may transfer electrons to CO_2_ to form active intermediates (CO_2_^-^), which are the main reactive species for the further reaction to generate CO.

### 3.3 Preparation and properties of C_3_N_4_/Au/SA hydrogel

**Fig. 3:**
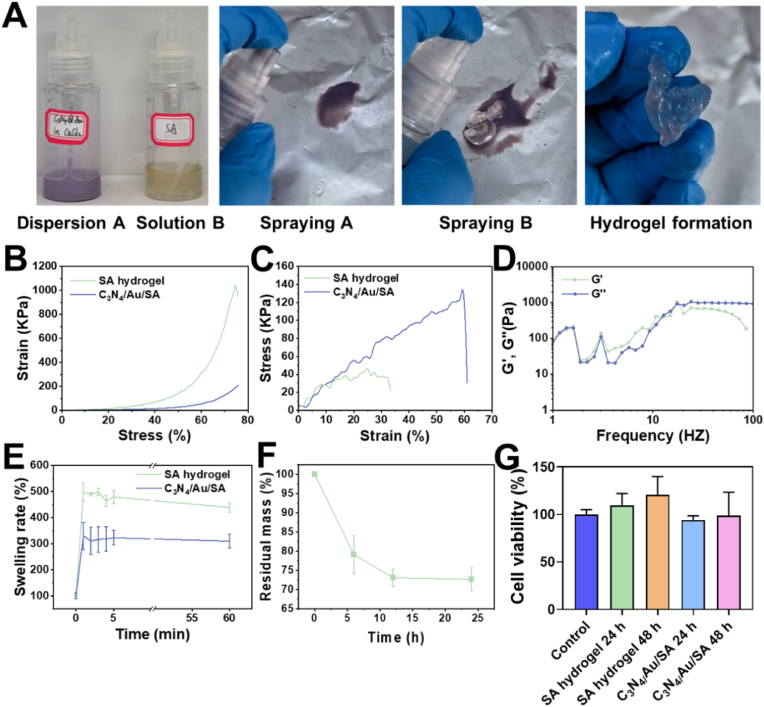
Spray-forming and characterization of C_3_N_4_/Au/SA hydrogels. **A)** Images of the process of C_3_N_4_/Au/SA hydrogel formation via spraying. **B)** Compression curves of the hydrogels. **C)** Tensile curves of the hydrogels. **D)** Rheology curve of C_3_N_4_/Au/SA hydrogel. **E)** Swelling ratios of the hydrogels. **F)** degradation curve of C_3_N_4_/Au/SA hydrogel. **G)** Cytocompatibility of the hydrogels with L929 cells after incubation for various durations.

To ensure full coverage of wounds after tumor removal, SA and CaCl_2_ were dissolved separately in two spray bottles, allowing for quick hydrogel film formation through spraying due to their rapid cross-linking abilities. Although C_3_N_4_/Au could be dispersed in either SA or CaCl_2_ solution, we opted to disperse it in the less viscous CaCl_2_ solution to achieve even distribution using ultrasonication. As shown in Fig. 3A, the two hydrogel precursors are illustrated: C_3_N_4_/Au dispersion in CaCl_2_ solution (denoted as Dispersion A) and SA solution (denoted as Solution B). When sequentially sprayed onto a weighing paper, a film-like C_3_N_4_/Au/SA hydrogel formed immediately upon contact and could be easily removed.

Subsequently, the mechanical, rheological, swelling, and degradation properties of the C_3_N_4_/Au/SA hydrogel were investigated. As shown in Fig. 3B, both SA and C_3_N_4_/Au/SA hydrogels demonstrated excellent compressibility, maintaining structural integrity even when compressed beyond 70 % strain. The stress required to compress the C_3_N_4_/Au/SA hydrogel was less than 200 kPa, indicating the soft nature of C3N4/Au/SA hydrogel that could adapt to various surgical wound geometries. Tensile testing (Fig. 3C) revealed that while SA hydrogel fractured at 34 % strain, C_3_N_4_/Au/SA hydrogel could withstand over 60 % strain, suggesting that C_3_N_4_/Au incorporation enhanced flexibility to accommodate patient mobility during wound management. Rheological characterization (Fig. 3D) showed that the storage modulus (G′) slightly exceeded the loss modulus (G″) at low frequencies, while G″ surpassed G′ at high frequencies, indicating the C_3_N_4_/Au/SA hydrogel existed near the gelation point-a characteristic feature of ionically cross-linked hydrogels that enables rapid formation and mild self-healing properties.

Fig. 3E showed the swelling properties of the hydrogels. The SA hydrogel had a strong water-absorption capacity and reached the swelling equilibrium within 1 min after absorbing water, with a swelling ratio of 490 %. After adding C_3_N_4_/Au, probably due to the introduction of hydrophobic components, the swelling ratio of the C_3_N_4_/Au/SA hydrogel decreases to 330 %. However, the rate of water absorption remained constant, and the system achieved swelling equilibrium within 1 min. The release of components from C_3_N_4_/Au within the hydrogel was evaluated through hydrogel degradation. The degradation curve of the C_3_N_4_/Au/SA hydrogel in water was shown in Fig. 3F. Within 24 h, the mass of the hydrogel decreased by 27 %, indicating that the hydrogel can slowly degrade upon contact with body fluids and release the C_3_N_4_/Au within it, so as to more effectively catalyze the release of CO near the wound surface under irradiation.

The cell compatibility of the hydrogels was verified by co-culturing normal cells with the hydrogel leachate and C_3_N_4_/Au. As shown in Fig. 3G, after co-culturing L929 cells with the leachate of SA hydrogel and C_3_N_4_/Au/SA hydrogel for 24 and 48 h, the cell viability was over 94 %. This indicated that both the SA hydrogel and the nanoparticle-loaded hydrogel have good biosafety. In addition, the cytotoxicity of g-C_3_N_4_ and C_3_N_4_/Au was shown in Fig. S11. It can be observed that g-C_3_N_4_ and C_3_N_4_/Au at concentrations within 200 μg/mL did not cause a significant decrease in the viability of L929 cells, indicating that the materials have good cytocompatibility.

### 3.4 In vitro CO generation from C_3_N_4_/Au and scratch closure assay

**Fig. 4:**
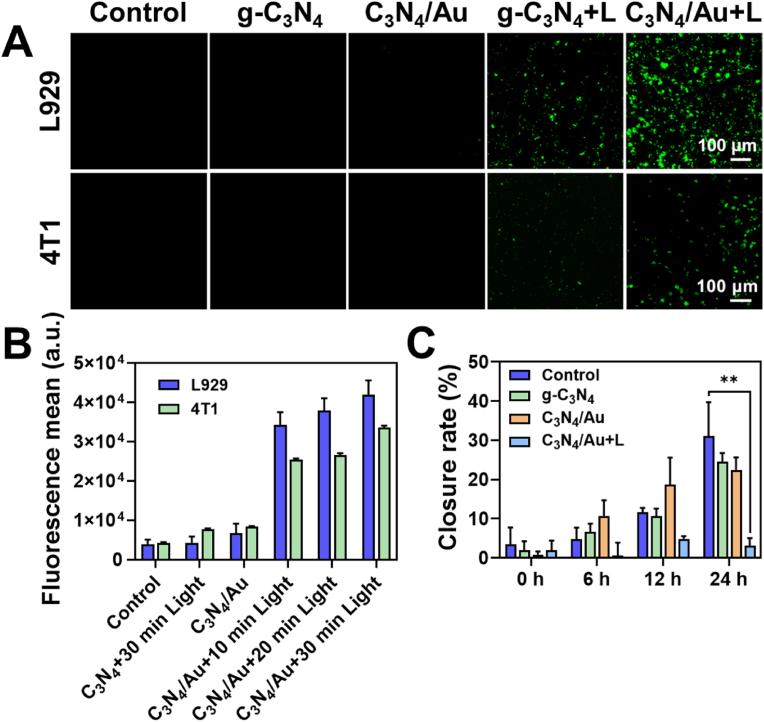
The ability of C_3_N_4_/Au to generate CO within cells and inhibit the invasion of tumor cells. **A)** Fluorescence images of CO generation before and after light exposure after incubation of g-C_3_N_4_ and C_3_N_4_/Au with L929 and 4T1 cells. **B)** The mean fluorescence intensity of LysoFP-NO_2_ in L929 and 4T1 cells after culturing with g-C_3_N_4_ and C_3_N_4_/Au measured by flow cytometry. **C)** scratch closure rates of 4T1 cells after culturing with g-C_3_N_4_ and C_3_N_4_/Au at different durations.

Further assessment was conducted to evaluate the in vitro CO production capability of C_3_N_4_/Au in L929 and 4T1 cells using the CO fluorescent probe LysoFP-NO_2_. As depicted in Fig. 4A, under non-illuminated conditions, no fluorescence signals were observed for either the C_3_N_4_ or the C_3_N_4_/Au group. However, upon light exposure, the C_3_N_4_ group exhibited a relatively weak green fluorescence signal, indicative of its capacity to reduce CO_2_ to CO when excited by blue light. In comparison, the C_3_N_4_/Au group under light exposure demonstrated a substantial increase in green fluorescence, signifying CO generation. This enhancement in fluorescence further illustrates the effectiveness of Au doping in improving the catalytic efficiency of C_3_N_4_ for the reduction of CO_2_ to CO.

Next, the intracellular CO concentration of L929 and 4T1 cells was quantitatively analyzed using a flow cytometer. As shown in Fig. 4B, negligible fluorescence was observed in the cells of the control group without treatment, the g-C_3_N_4_ group subjected to 30 min of light exposure, and the C_3_N_4_/Au group without light exposure. Conversely, upon light irradiation of the C_3_N_4_/Au group, the fluorescence intensity of LysoFP-NO_2_ within the cells progressively increased over time. This observation indicated that the photocatalytically generated CO was able to diffuse freely into the cells. Following 30 min of light exposure, the average fluorescence intensities in L929 and 4T1 cells were 10.9-fold and 7.9-fold higher compared to the control group, respectively. This finding suggested that C_3_N_4_/Au is capable of generating CO in both normal and tumor cells.

The in vitro scratch closure assay was used to evaluate the ability of the material to generate CO and inhibit the invasion of tumor cells. The 4T1 cells were scratched and then treated with g-C_3_N_4_ or C_3_N_4_/Au. The invasion of tumor cells was evaluated according to the proportion of scratch closure. As shown in Fig. 4C and Fig. S6, both g-C_3_N_4_ and C_3_N_4_/Au led the cells to migrate towards the gap without light irradiation. At the 24 h time-point, it was observed that the scratch in the control group closed by 31 %, while those in the g-C_3_N_4_ and C_3_N_4_/Au groups closed by 24 % and 22 % respectively, showing no significant difference from the control group. In contrast, the scratch in the C_3_N_4_/Au + light group closed by only 3 %, which was significantly lower than that in other groups. This indicated that CO had the ability to resist the migration of tumor cells, reducing the probability of tumor metastasis and invasion after surgery.

### 3.5 In vitro chemosensitization effect of CO generation

**Fig. 5:**
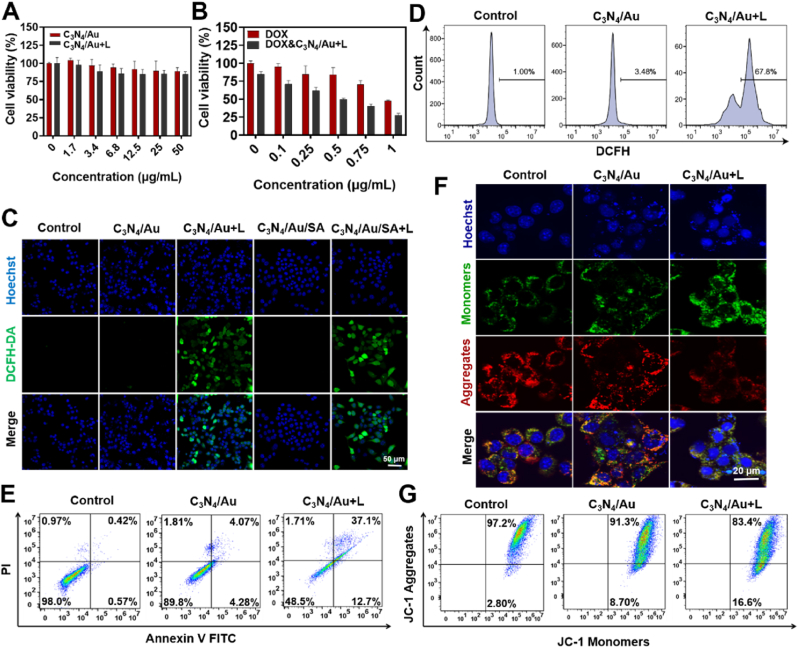
*In vitro* promoting effects on cell apoptosis and chemotherapy of photocatalytic reduction of C_3_N_4_/Au to generate CO. **A)** The cytotoxicity of C_3_N_4_/Au and C_3_N_4_/Au + Light against 4T1 cells. **B)** The cytotoxicity of DOX and DOX + C_3_N_4_/Au + Light against 4T1 cells. **C)** Imaging of ROS production within 4T1 cells treated with C_3_N_4_/Au and C_3_N_4_/Au/SA under irradiation. **D)** Flow cytology of ROS production in 4T1 cells treated with C_3_N_4_/Au under irradiation. **E)** Flow cytology of early and late apoptosis of 4T1 cells treated with C_3_N_4_/Au under irradiation. **F)** Imaging and **G)** flow cytology of mitochondrial membrane potential of 4T1 cells treated with C_3_N_4_/Au under irradiation.

After confirming the ability of C_3_N_4_/Au to catalyze the reduction of CO_2_ to CO within cells under blue light irradiation, the impact of CO gas therapy in synergy with DOX on the cell viability of 4T1 cells was investigated using the CCK-8 assay. The cell viability of C_3_N_4_/Au was first evaluated under different concentrations and conditions. As shown in Fig. 5A, no significant cytotoxicity was observed for C_3_N_4_/Au when the concentration ranged from 0 to 50 μg/mL, regardless of whether it was under light exposure or not.

The employment of the amphiphilic block copolymer DSPE-PEG for drug loading resulted in the successful preparation of DM. The modification of DM with FA-conjugated DSPE-PEG led to the fabrication of FA@DM. Fluorescence quantification analysis revealed a drug loading efficiency of 8.24 % for FA@DM, along with an encapsulation efficiency of 44.9 % (Table S1). TEM image displayed a uniform spherical structure for FA@DM (Fig. S7). The hydrodynamic particle size of FA@DM increased slightly from 8.3 nm to 17.66 nm compared to unmodified DM (Fig. S8). Zeta potential measurements indicated that the FA modification significantly decreased the surface charge of DM from -20.02 mV to -40.81 mV, suggesting that the introduction of FA notably enhanced the negative surface charge of DM (Fig. S9). The drug release profiles showed that DOX exhibited a sustained release behavior from both FA@DM and DM. After 24 h, the drug release rate reached approximately 70 % for both formulations (Fig. S10), indicating that FA@DM and DM possessed favorable drug release properties.

Subsequently, a progressive decrease in the cell viability of 4T1 cells was observed when the concentration of C_3_N_4_/Au was maintained at 50 μg/mL and the concentration of FA@DM increased. Additionally, under identical DOX concentrations, 4T1 cells treated with CO displayed heightened sensitivity to DOX. Specifically, under the influence of CO, the viability of 4T1 cells was approximately 70 % at a DOX concentration of 0.1 μg/mL. In contrast, without CO stimulation, a DOX concentration of 0.75 μg/mL was required to achieve a similar reduction in tumor cell viability. These results further confirm that CO can significantly enhance the sensitivity of tumor cells to DOX-induced cytotoxicity (Fig. 5B).

The next objective is to investigate the mechanism by which CO enhances the sensitivity of tumor cells to chemotherapy drugs. Some researchers suggest that CO influences cell behavior by promoting mitochondrial synthesis and increasing the production of reactive ROS [44,45]. In the context of tumor cells, the enhancement of mitochondrial function by CO may result in elevated ROS levels, which in turn can induce tumor cell apoptosis. Therefore, we verified the chemosensitization mechanism by detecting the effects of CO generated by photocatalysis of C_3_N_4_/Au on ROS and mitochondrial membrane potential in tumor cells. To evaluate ROS production within cells under CO stimulation, a ROS probe, DCFH-DA was employed. Fig. 5C illustrated that the C_3_N_4_/Au + L group exhibited a significant green fluorescence, indicative of ROS production, when compared to the Control and C_3_N_4_/Au groups. This observation indicates that CO generated by C_3_N_4_/Au under blue light stimulation indeed stimulates ROS production within cells. Additionally, the capacity of C_3_N_4_/Au/SA hydrogel to stimulate ROS production in cells was tested. It was found that, under light stimulation, C_3_N_4_/Au/SA can also induce a substantial increase in ROS within cells. The intracellular ROS production was quantitatively analyzed by flow cytometry, as shown in Fig. 5D. After the addition of C_3_N_4_/Au, only a very small number of cells (3.48 %) had green fluorescence, while this proportion increased to 67.8 % after light exposure, and the average fluorescence intensity is 12.87 times that of the control group (Fig. S12). This further confirmed that C_3_N_4_/Au could increase the intracellular oxidative stress level after light exposure.

Further analysis was conducted on tumor cells treated under various conditions using PI/Annexin-V double staining and flow cytometry. Fig. 5E and Fig. S13 illustrates that tumor cells treated with C_3_N_4_/Au and exposed to light stimulation showed significantly higher rates of early apoptosis (12.7 %) and late apoptosis (37.1 %) compared to the other groups. These findings further confirm the sensitivity of tumor cells to oxidative stress induced by ROS, leading to the induction of apoptotic states and, consequently, enhancing the sensitivity to chemotherapy drugs.

The JC-1 Mitochondrial Membrane Potential Assay Kit was utilized to examine the alterations in mitochondrial membrane potential within tumor cells under different conditions. Fig. 5F illustrates that the Control group, as well as the C_3_N_4_/Au group, showed a pronounced bright red fluorescence from J-aggregates, signifying a high mitochondrial membrane potential. Conversely, the C_3_N_4_/Au + L group exhibited a notable enhancement in green fluorescence. This observation suggests that CO generated by C_3_N_4_/Au under blue light irradiation can diminish the mitochondrial membrane potential, prompting a shift in JC-1 from the aggregate to the monomer state. Flow cytometry was subsequently used to validate the proportion of cells in the monomer state, which rose from 2.8 % in the Control group and 8.7 % in the C_3_N_4_/Au group to 16.6 % in the C_3_N_4_/Au + L group (Fig. 5G and Fig. S14). In summary, C_3_N_4_/Au is capable of producing CO upon stimulation by blue light irradiation, which in turn can lead to a reduction in the mitochondrial membrane potential of tumor cells. This reduction results in the generation of substantial ROS, thereby triggering the cells to undergo apoptosis. Consequently, the tumor cells become more sensitive to chemotherapy drugs.

### 3.6 Tumor-targeting effects of FA@DM in vitro and in vivo

**Fig. 6:**
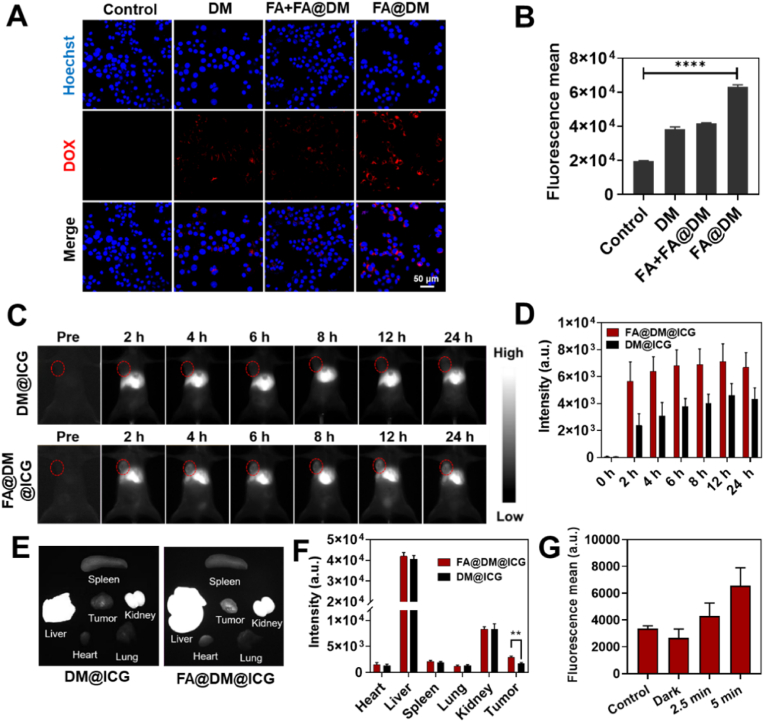
The tumor targeting effect of FA@DM. **A)** The cellular uptake of DOX and FA@DM in 4T1 cells. **B)** Flow cytometry fluorescent quantification of DOX in in 4T1 cells. **C)** NIR-II imaging of intratumoral DOX retention at different times after intravenous injection. **D)** Quantitative fluorescence intensity of tumor at different times. **E)** NIR-II imaging of heart, liver, spleen, lung, kidney and tumor 24 h after injection of DM and FA@DM. **F)** Quantitative fluorescence intensity of each tissue. **G)** Mean fluorescence intensity of LysoFP-NO_2_ within the tumors of mice after surgery measured by flow cytometry.

Next, the uptake of FA@DM by 4T1 cells was observed using CLSM. Fig. 6A shows that the FA@DM group exhibited stronger red fluorescence compared to the DM group, indicative of greater uptake by tumor cells. This suggests that FA@DM, modified with FA, has enhanced targeting capabilities for 4T1 tumor cells. Concurrently, FA pre-treatment was utilized to block the FA receptors on the surface of 4T1 cells. Upon comparison, the red fluorescence in the FA pre-treated group (FA + FA@DM) was significantly weaker than that in the FA@DM group. This finding further demonstrates that the FA receptor plays a key role in the increased uptake of FA@DM by 4T1 cells and reaffirms that the modification with FA significantly enhances the tumor targeting of FA@DM. The targeting performance of FA@DM was also characterized using flow cytometry, with the results shown in Fig. 6B further validating the tumor-targeting effect of FA.

Furthermore, to accurately observe the distribution of micelles in vivo, micelles was labeled with the NIR-II dye ICG. This can avoid autofluorescence and endow stronger tissue-penetrating ability. FA@DM and DM, modified with ICG, were administered intravenously *via* the tail vein to mice, resulting in FA@DM@ICG and DM@ICG, respectively. The in vivo distribution of these formulations was explored using NIR-II in vivo imaging system. Fig. 6C demonstrates that whereas the DM@ICG group exhibited a weak fluorescence signal in the tumor region, a distinct signal was observed in the tumor region of mice injected with FA@DM@ICG as early as 2 h post-injection, which persisted for up to 24 h. Quantitative fluorescence data from the tumor region indicated that the fluorescence signal in the FA@DM@ICG tumor region was consistently higher than that in the DM@ICG group at the same time points (Fig. 6D). After 24 h of intravenous injection, fluorescence imaging of the major tissues revealed that most micelles were metabolized through the liver and kidneys (Fig. 6E). The FA@DM@ICG group, in comparison to the DM@ICG group without FA modification, showed stronger fluorescence signals in tumor tissues (Fig. 6F). These results further confirm the in vivo tumor targeting efficacy of FA and provide a basis for the application of FA@DM in drug delivery post-tumor surgery.

In addition, we also confirmed whether C_3_N_4_/Au/SA could photocatalytically generate CO in vivo. As shown in Fig. 6G and Fig. S15, the CO in the post-surgical tumors of mice after photocatalysis was detected using the CO fluorescent probe LysoFP-NO_2_, and quantified by flow cytometry. It was found that the CO concentration increased with the illumination time, indicating that the CO generated by the photocatalysis of C_3_N_4_/Au/SA could diffuse into the residual tumors, facilitating the subsequent verification of the anti-tumor effect.

### 3.7 *In vivo* anti-tumor effect of CO sensitizes chemotherapy

**Fig. 7:**
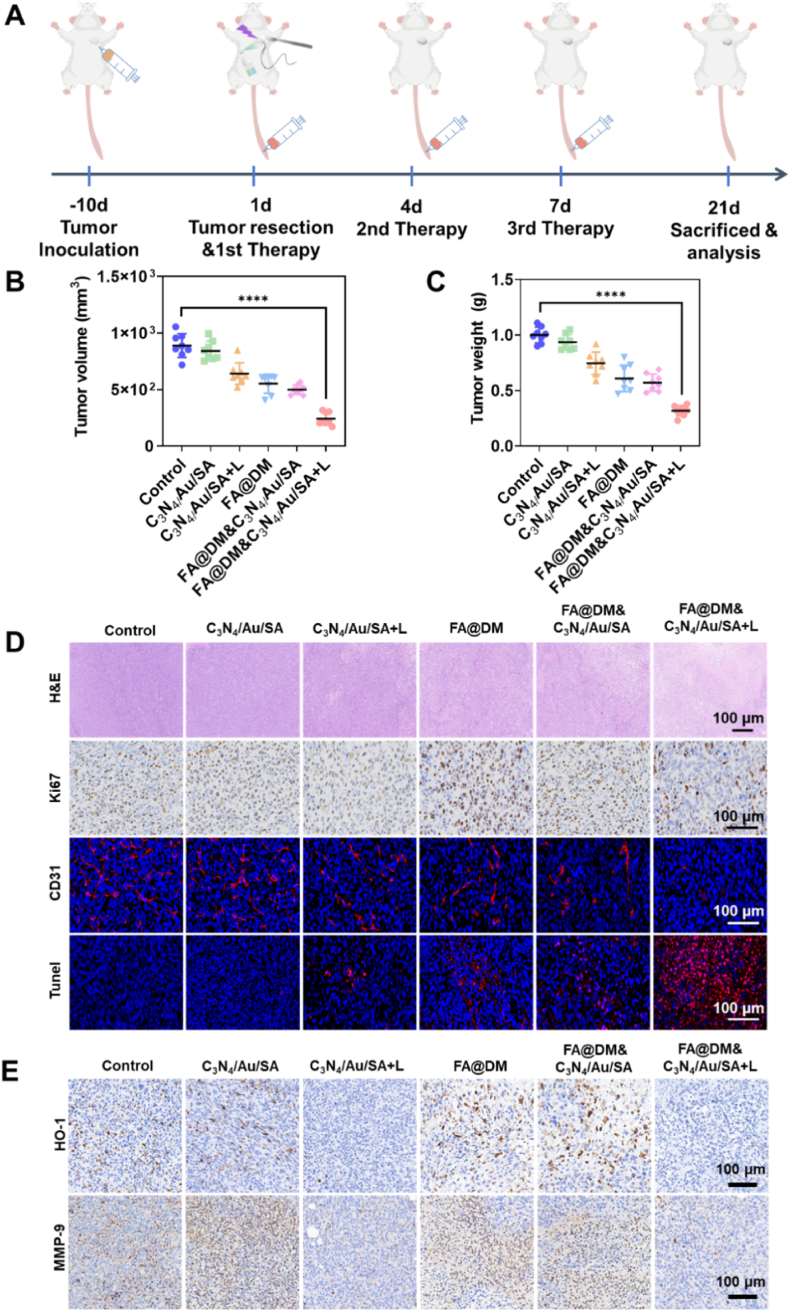
**A)** Diagram of in vivo experiments. **B)** Tumor volume and **C)** tumor weight at 21 days after treatment of different groups. **D)** Sections of tumor tissues stained by H&E, Ki67 immunohistochemistry, CD31 Immunofluorescence and Tunel at 21 days after treatment of different groups. **E)** Sections of tumor tissues stained by HO-1 and MMP-9 immunohistochemistry at 21 days after treatment of different groups.

The postoperative breast cancer model was constructed, followed by an evaluation of the antitumor effects of C_3_N_4_/Au/SA generating CO synergistic FA@DM over a 21-day treatment period. As depicted in Fig. 7A, orthotopic modeling of breast cancer in mice was established initially. When the tumor volume reached approximately 100 mm^3^, tumor resection was performed in situ, removing about 80 % of the tumor volume. A solution containing calcium chloride and C_3_N_4_/Au was sprayed onto the surgical wound, followed by a solution of SA. Once the hydrogel had formed, the wound was exposed to blue light for 5 min. Following this, the drug was administered intravenously on the 1st, 4th, and 7th days post-surgery, concluding the 21-day treatment period. As shown in Fig. 7B, following the conclusion of a 21-day treatment, tumor tissues from the mice were excised and observed. Both the FA@DM group and the C_3_N_4_/Au/SA + L group demonstrated slightly better antitumor effects compared to the Control group. These results suggest that apoptosis and cell death within the tumor, either triggered by CO or induced by the chemotherapy drug DOX, can lead to a certain degree of tumor growth suppression. However, the combination of C_3_N_4_/Au/SA + L with FA@DM significantly enhanced the inhibition of tumor growth. Similarly, after the 21-day treatment period, tumor tissues from the mice were excised and weighed. The results indicated that the lowest weight of tumor tissues was found in the FA@DM&C3N4/Au/SA + L group (Fig. 7C). Furthermore, photographs taken after the excision of tumor tissues from each group demonstrated more directly the excellent antitumor effects of CO when synergized with chemotherapy (Fig. S16A).

As shown in Fig. S16B, throughout the 21-day treatment period, no significant changes in body weight were observed in mice across all groups. This indicates that neither the hydrogel C_3_N_4_/Au/SA nor the chemotherapeutic micelles FA@DM exhibited notable toxic side effects. Additionally, after the treatment, the major organs (heart, liver, spleen, lung, and kidney) of mice in each group were subjected to hematoxylin-eosin (H&E) staining. The results revealed no apparent tissue damage, further confirming this conclusion (Fig. S17). After the treatment, H&E and immunohistochemical/fluorescence staining were performed on the tumor tissues of mice in each group, with the results displayed in Fig. 6D. H&E staining revealed dense, dark purple, abnormally proliferating cell nuclei and numerous mitotic figures in the Control and C_3_N_4/_Au/SA groups, indicating an abnormally rapid proliferation rate in these tumor tissues. In contrast, areas of apoptosis and necrosis were observed in the other four groups. Notably, extensive necrotic areas were evident in the FA@DM&C_3_N_4_/Au/SA + L group. Correspondingly, TUNEL staining sections of tumor cells showed a large amount of red fluorescence representing cell apoptosis in the FA@DM&C3N4/Au/SA + L group, further indicating that CO gas stimulation combined with FA@DM chemotherapy significantly enhanced tumor cell apoptosis. Immunohistochemical staining for Ki-67 was also conducted on the tumor tissues. The results indicated that the content of Ki-67-positive cells in the FA@DM&C_3_N_4_/Au/SA + L group was significantly lower than in the other groups, suggesting that CO in combination with chemotherapy significantly inhibited tumor cell proliferation. Another interpretation is that CO may cause cell cycle arrest at certain phases, such as G0/G1 or G2/M phases which can increase the cytotoxic effect of chemotherapeutic drugs on tumor cells. Furthermore, comparison of CD31 fluorescence staining results revealed that CO can inhibit tumor angiogenesis, effectively limiting the acquisition of nutrients by tumor cells and thereby restricting the tumor growth environment. Combined with chemotherapeutic drugs, this significantly enhances the antitumor effect.

The potential mechanisms by which CO enhanced therapeutic effects in vivo were ultimately explored and elucidated through alterations in downstream cytokine levels induced by CO exposure. The expression levels of heme oxygenase-1 (HO-1) and matrix metalloproteinase-9 (MMP-9) in mouse tumors in each group after treatment were analyzed by immunohistochemistry. CO was found to induce the overexpression of ROS in macrophages, activate the MAPK/Erk1/2-cmyc signaling pathway and down-regulate the Notch 1-dependent HO-1 expression [46]. As shown in Fig. 6E, following the application of light to the C_3_N_4_/Au/SA hydrogel, a significant downregulation of HO-1 expression was observed, suggesting that CO effectively penetrated into the tumor tissue. when the combined treatment of FA@DM and the C_3_N_4_/Au/SA hydrogel was administered with light, a marked downregulation of HO-1 expression was noted compared to the control group or the group receiving only FA@DM. Combining with the results of the smallest tumor volume and mass of FA@DM&C_3_N_4_/Au/SA + L group in Fig. 6B and C, it is proved that CO caused an increase in ROS and had a synergistic effect with DOX to significantly inhibit tumor growth.

A positive correlation exists between the expression level of MMP-9 and tumor invasion [47]. As shown in Fig. 6E, the expression level of MMP-9 in the C_3_N_4_/Au/SA + light group was significantly down-regulated, indicating that CO can inhibit tumor invasion, which is consistent with previous reports [48]. In addition, the expression level of MMP-9 after the combined treatment of CO and DOX was significantly lower than that observed with DOX treatment alone. This exhibited that CO can inhibit the invasion of tumor cells stimulated during DOX treatment, thereby enhancing the efficacy of post-surgical chemotherapy.

## 4 Conclusion

In summary, an innovative sprayable composite hydrogel drug delivery system has been successfully developed, opening up a new, safe, and highly efficient pathway for postoperative tumor treatment. When the nanophotocatalyst C_3_N_4_/Au is sprayed at the surgical incision site, a hydrogel can be rapidly formed in situ. Under visible light irradiation, C_3_N_4_/Au efficiently converts CO_2_ in the tumor region into CO. That C_3_N_4_/Au has a strong ability to generate CO has been confirmed by in vitro experiments. The mitochondrial membrane potential is affected, leading to an increase in the production of ROS in tumor cells, which in turn enhances the sensitivity of tumor cells to the chemotherapeutic drug doxorubicin (DOX). Enhanced anti-tumor effects against breast cancer cells are demonstrated by the combination of CO stimulation and DOX. Tumor growth is significantly inhibited and tumor cell apoptosis and necrosis are enhanced by the combination of CO generation and FA@DM chemotherapy, as indicated by in vivo experimental results. This combined treatment effectively inhibits tumor cell proliferation and angiogenesis is further confirmed by histological staining. New insights into the construction of gas therapy and efficient chemotherapy strategies in postoperative tumor treatment are provided by this system.

Ethics approval and consent to participate

All experiments and animal procedures were approved by the Ethical Committee of Shanghai University of Medicine & Health Sciences Affiliated Zhoupu Hospital and conducted in accordance with the Guide for Care and Use of Laboratory Animals (approval number: ZPYYLL-2018-02).

## CRediT authorship contribution statement

**Zaiyan Wang:** Writing – original draft, Investigation, Conceptualization. **Jianxiang Zhu:** Writing – original draft, Investigation, Conceptualization. **Bobin Mi:** Methodology, Investigation, Conceptualization. **Ming Ni:** Writing – original draft, Methodology, Conceptualization. **Yuming Xue:** Investigation, Formal analysis, Data curation. **Yiling Deng:** Investigation, Formal analysis, Data curation. **Lu Chen:** Investigation, Formal analysis, Data curation. **Xiangyang Xu:** Writing – review & editing, Supervision. **Xiaoyan Li:** Writing – review & editing, Supervision. **Guohui Liu:** Writing – review & editing, Supervision. **Tao Yu:** Writing – review & editing, Supervision.

## Declaration of competing interest

The authors declare no conflict of interest.
