# Supplementary material for: Photocatalytic CO-releasing spray hydrogel for in situ postoperative cancer treatment
Source: Bioact Mater. 2025 Aug 12;53:893–907. doi: 10.1016/j.bioactmat.2025.07.024 (PMC12362012; doi:10.1016/j.bioactmat.2025.07.024)
Supplement: Multimedia component 1 [file mmc1.docx]

**Photocatalytic CO-releasing Spray Hydrogel for In Situ Postoperative Cancer Treatment**

**Table S1**. Drug encapsulation ratio and drug loading ratio of DM and FA@DM.

**Table S2**. Elemental mass fractions of C_3_N_4_/Au measured by XPS.

**Fig. S1**. Zeta potentials of g-C_3_N_4_ and C_3_N_4_/Au.

**Fig. S2**. Synthesis Route and the CO response reaction of LysoFP-NO_2_.

**Fig. S3**. ^1^H NMR spectrum of LysoFP-NO_2_.

**Fig. S4**. HRMS of LysoFP-NO_2_.

**Fig. S5**. Hemolysis ratio of A) g-C_3_N_4_ and B) C_3_N_4_/Au.

**Fig. S6**. Images of the 4T1 cells scratch after different treatment.

**Fig. S7.** TEM image of FA@DM.

**Fig. S8**. The hydrodynamic particle size of FA@DM.

**Fig. S9**. The zeta potential of FA@DM.

**Fig. S10.** The ratio of DOX released from DM or FA@DM in 24 h.

**Fig. S11**. The cytocompatibility of g-C_3_N_4_ and C_3_N_4_/Au with L929 cells.

**Fig. S12**. Flow cytometry average fluorescence intensity of DCFH-DA in 4T1 cells.

**Fig. S13**. Flow cytometry average fluorescence intensity of apoptosis in 4T1 cells.

**Fig. S14**. Flow cytometry average fluorescence intensity of JC-1 in 4T1 cells.

**Fig. S15**. Flow cytometry analysis of CO generated by photocatalysis of C_3_N_4_/Au/SA in vivo under different illumination periods.

**Fig. S16**. Tumor photos and body weights of mice after treatment.

**Fig. S17**. H&E stained sections of the main organs of mice after 21 days of treatment.

**Table S1.** Drug encapsulation ratio and drug loading ratio of DM and FA@DM

|  | Encapsulation Ratio | Drug Loading Ratio |
| --- | --- | --- |
| DM | 46.22 | 10.66 |
| FA@DM | 44.9 | 8.24 |

**Table S2.** Elemental mass fractions of C3N4/Au measured by XPS

| Sample | Feed ratio of g-C_3_N_4_ to AuCl_3_ (g:mg) | Au% | C% | N% | O% |
| --- | --- | --- | --- | --- | --- |
| C_3_N_4_/Au-0.5 | 1:12.5 | 0.59 | 41.84 | 50.84 | 6.73 |
| C_3_N_4_/Au-1 | 1:25 | 0.89 | 41.59 | 51.17 | 6.35 |
| C_3_N_4_/Au-2 | 1:50 | 1.91 | 41.17 | 50.78 | 6.14 |


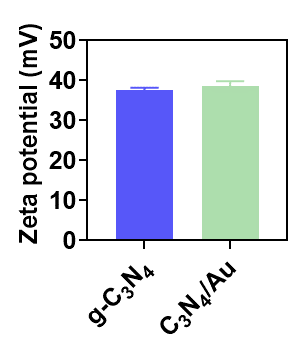


**Fig. S1**. Zeta potentials of g-C_3_N_4_ and C_3_N_4_/Au.


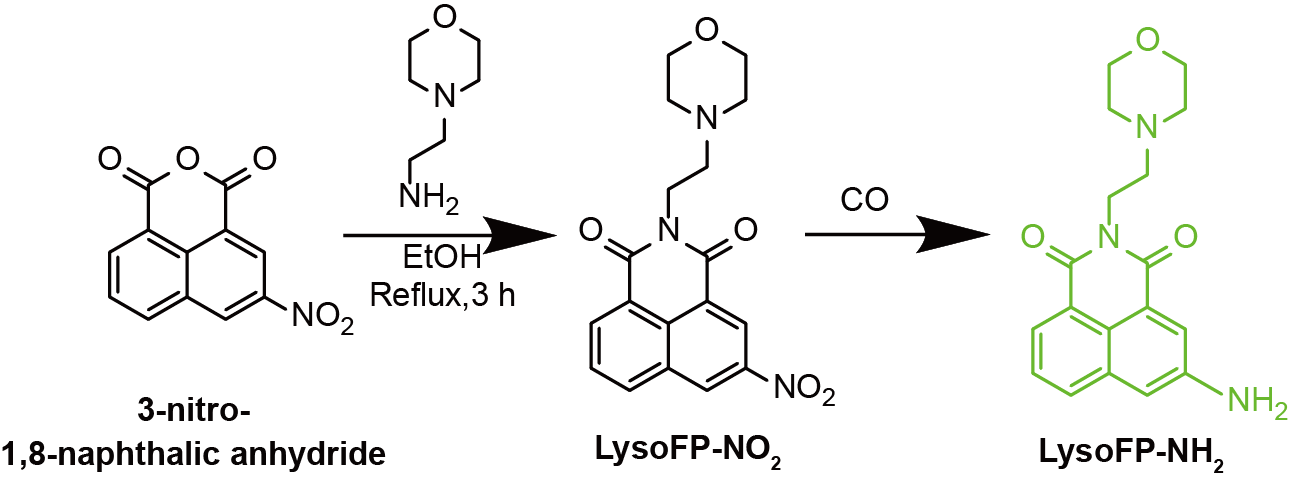


**Fig. S2**. Synthesis Route and the CO response reaction of LysoFP-NO_2_.


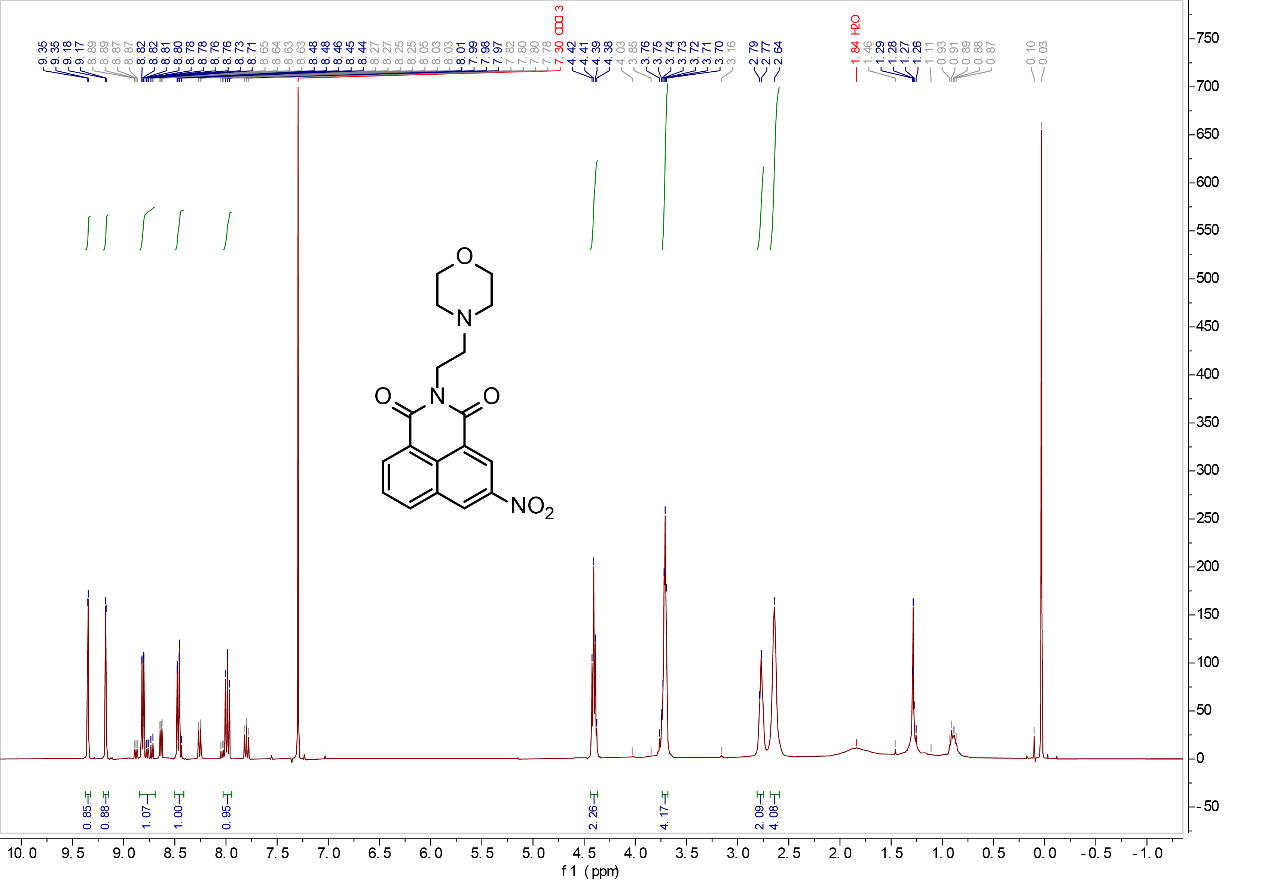


**Fig. S3**. ^1^H NMR spectrum of LysoFP-NO_2_.


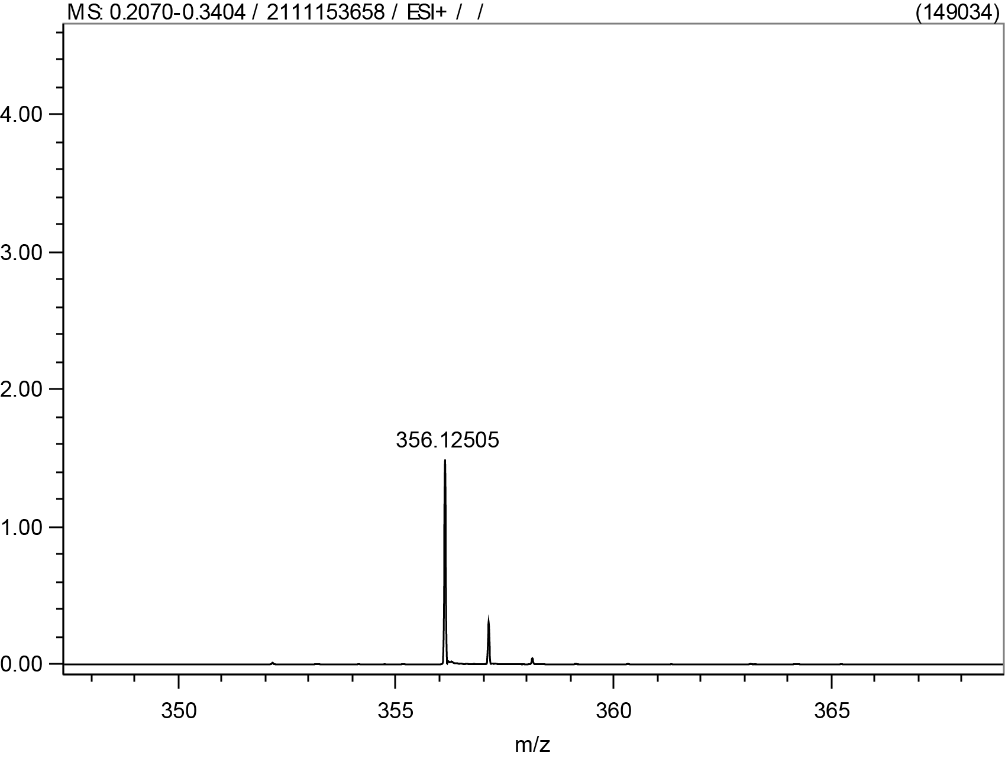


**Fig. S4**. HRMS of LysoFP-NO_2_.


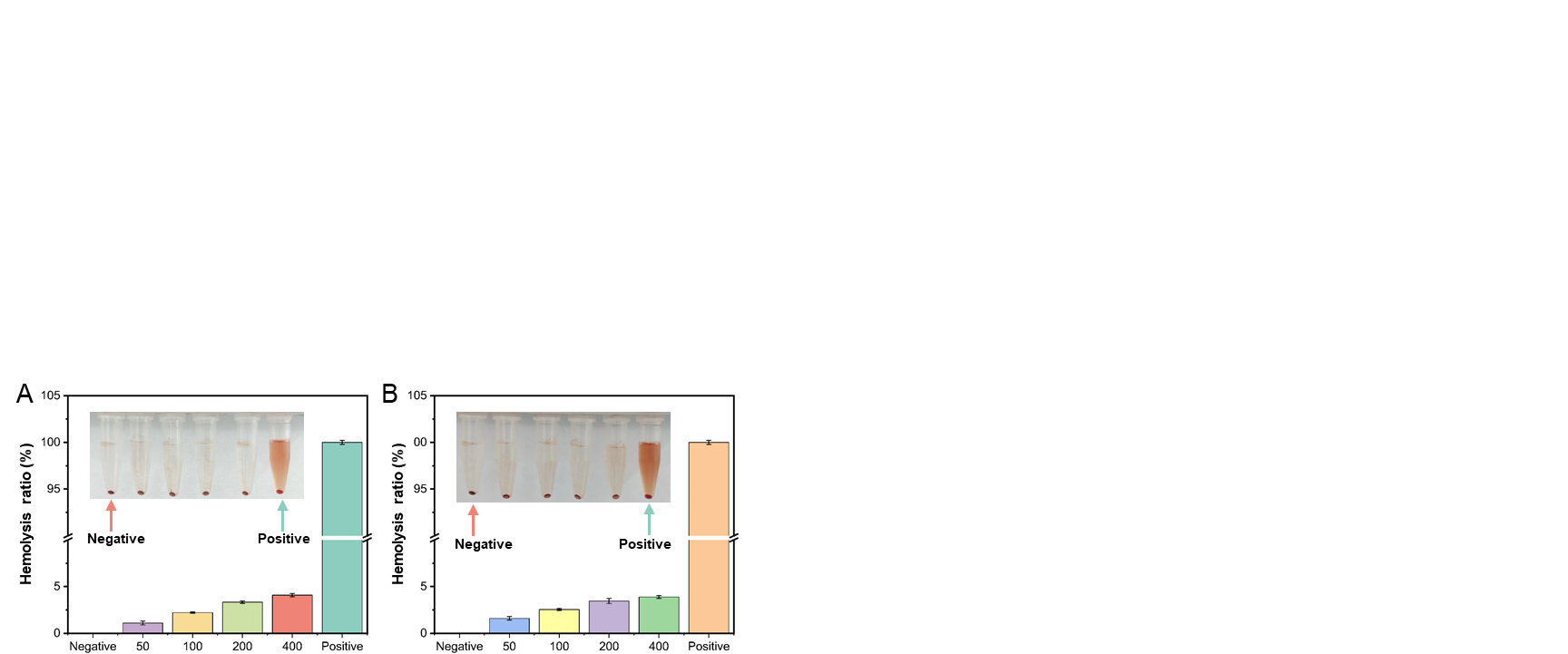


**Fig. S5**. Hemolysis ratios of A) g-C_3_N_4_ and B) C_3_N_4_/Au.


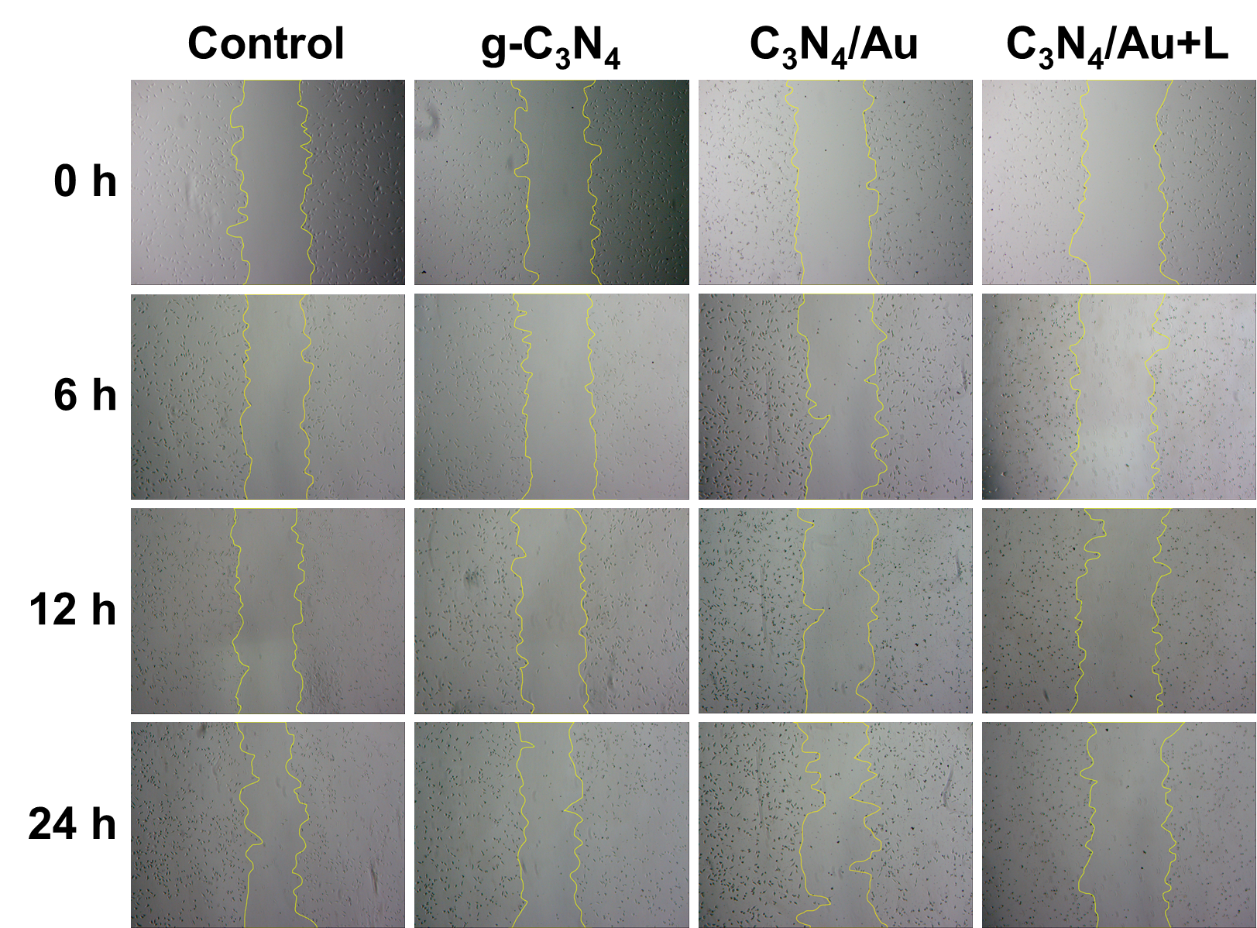


**Fig. S6**. Images of the 4T1 cells scratch after different treatment.


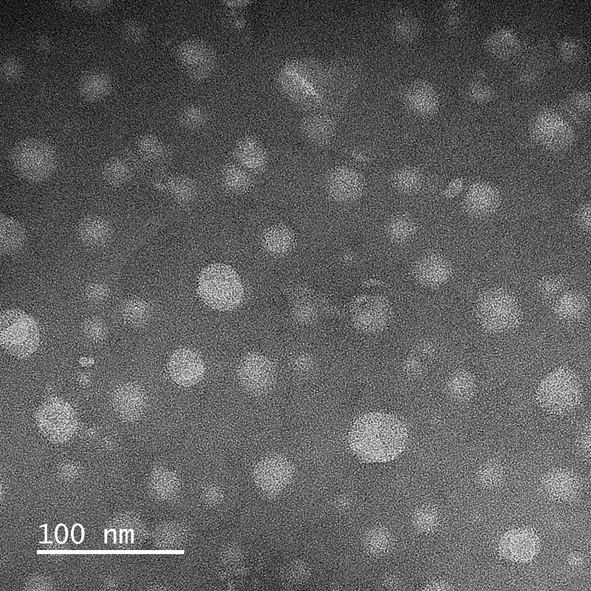


**Fig. S7**. TEM image of FA@DM

**Fig. S8**. The hydrodynamic particle size of FA@DM.

**Fig. S9**. The zeta potential of FA@DM.

**Fig. S10.** The ratio of DOX released from DM or FA@DM in 24 h.

**Fig. S11**. The cytocompatibility of g-C_3_N_4_ and C_3_N_4_/Au with L929 cells.

**Fig. S12**. Flow cytometry average fluorescence intensity of DCFH-DA in 4T1 cells treated with different materials.


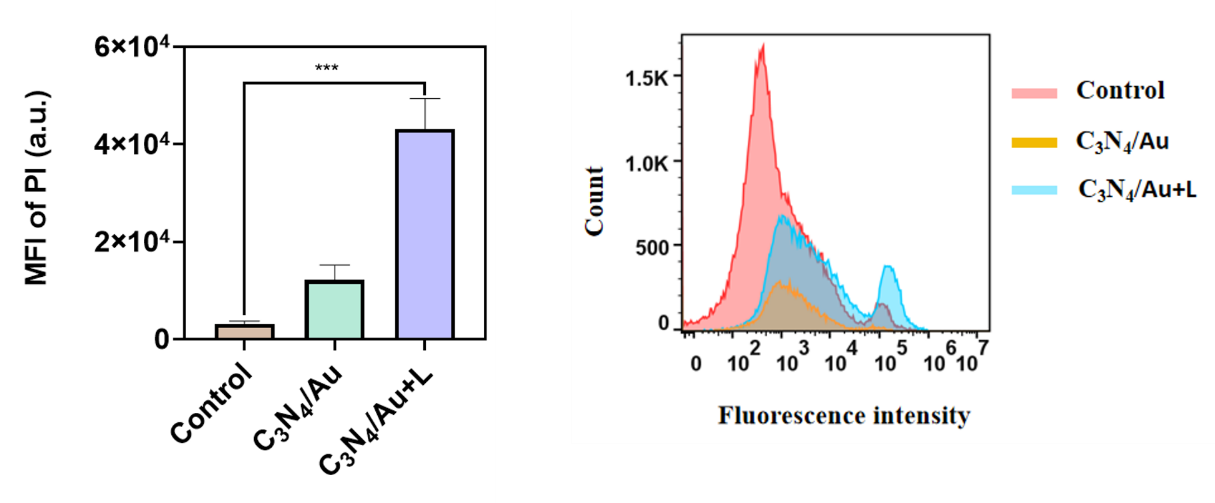


**Fig. S13**. Flow cytometry average fluorescence intensity of apoptosis in 4T1 cells.


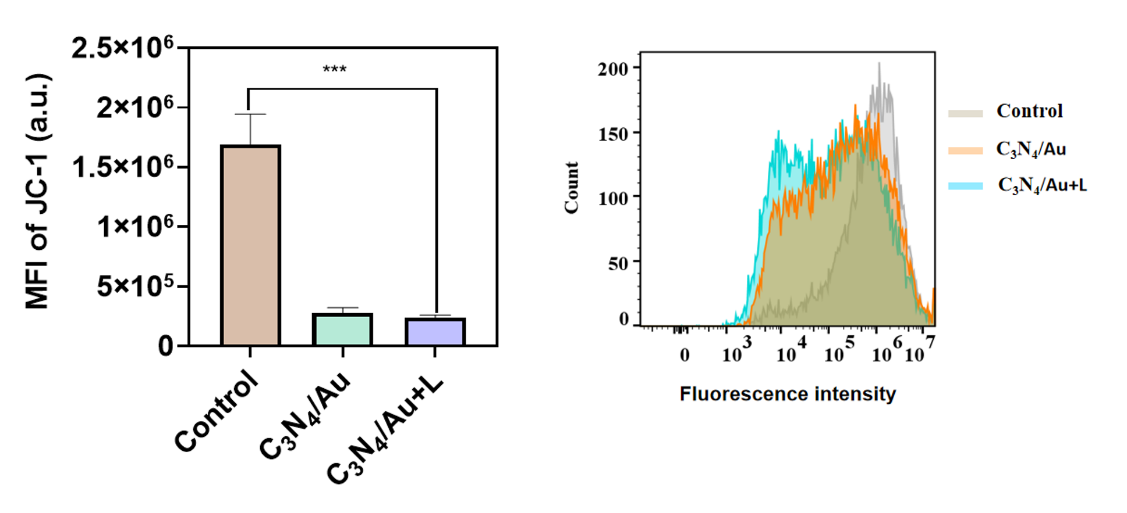
f

**Fig. S14**. Flow cytometry average fluorescence intensity of JC-1 in 4T1 cells.


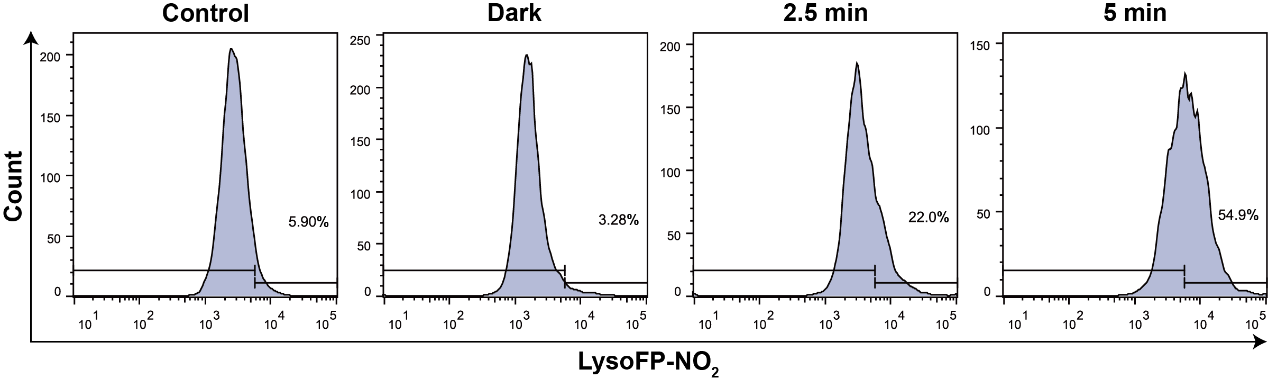


**Fig. S15**. Flow cytometry analysis of CO generated by photocatalysis of C_3_N_4_/Au/SA in vivo under different illumination periods.


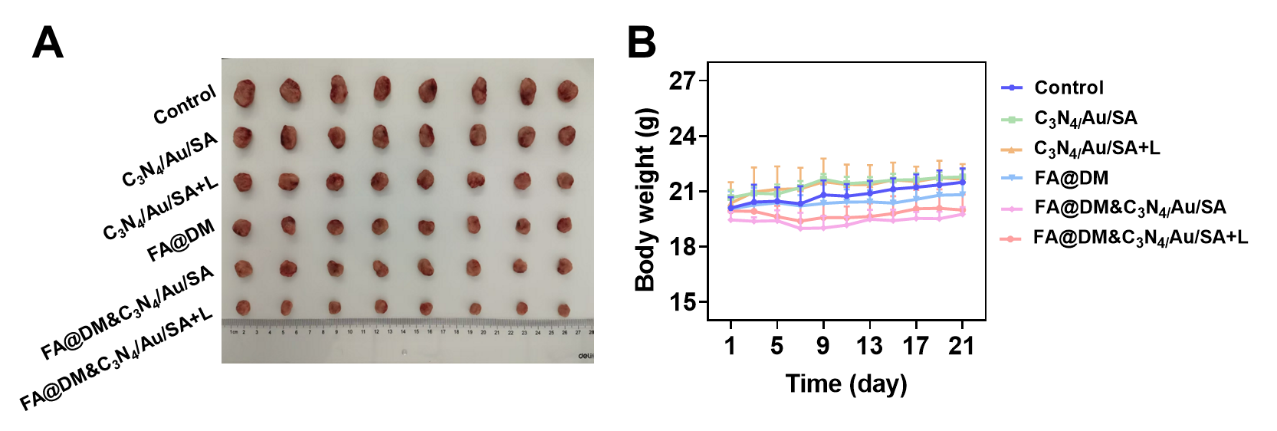


**Fig. S16**. Tumor photos and body weights of mice after treatment. A) Photographs of tumors at 21 days after treatment of different groups. B) Body weight changes of mouse during the treatment process.


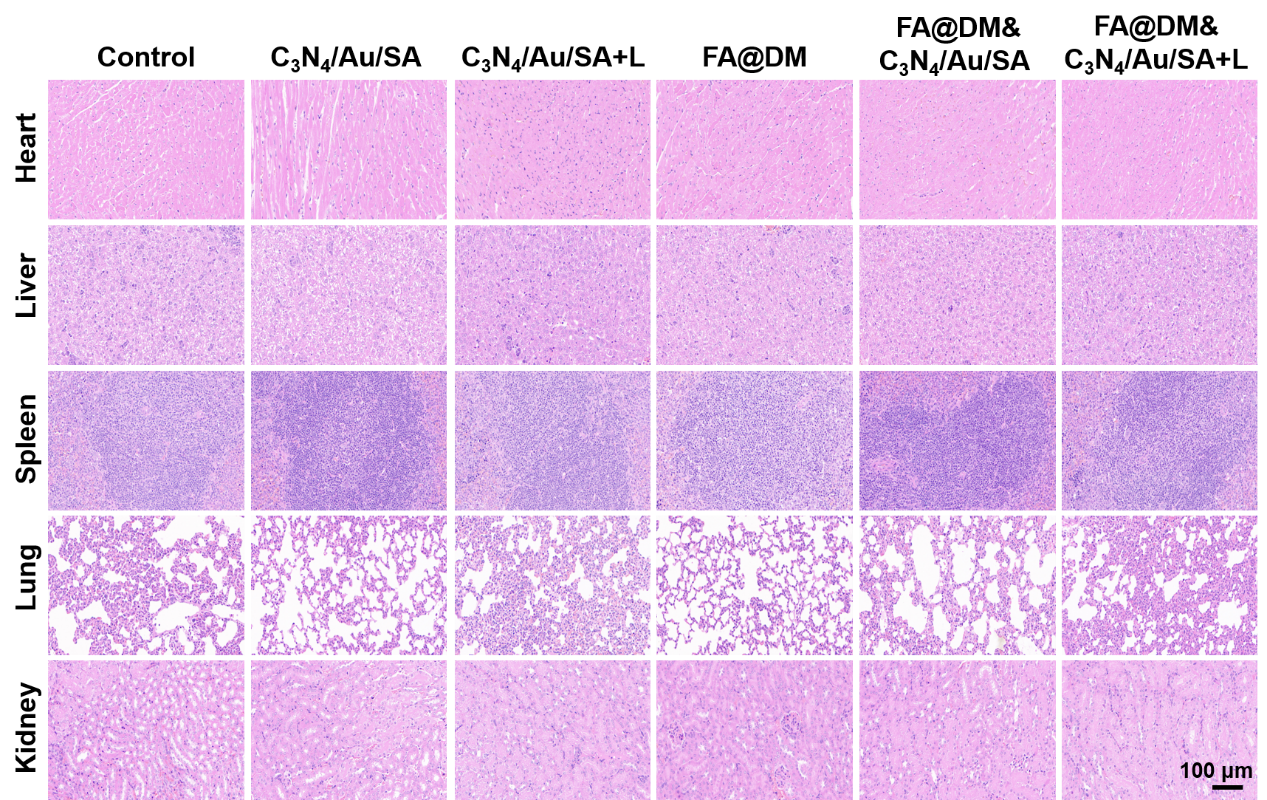


**Fig. S17**. Hematoxylin and eosin (H&E) stained sections of the heart, liver, spleen, lung, and kidney of mice after 21 days of treatment.
